# A novel vascular stent and insert concept to improve hemodynamics and support vascular health

**DOI:** 10.1038/s41598-025-09613-8

**Published:** 2025-07-08

**Authors:** Bahram Vaziri, Saadat Zirak, Mohammad Azadi, Amir Keshmiri, Nima Shokri

**Affiliations:** 1 Faculty of Mechanical Engineering, Semnan University, Semnan, Iran; 2 School of Engineering, The University of Manchester, Manchester, UK; 3 Manchester University NHS Foundation Trust, Manchester, M13 9PL UK; 4 Institute of Geo-Hydroinformatics, Hamburg University of Technology, Hamburg, Germany

**Keywords:** Stent, Insert, Hemodynamic, Pulsatile, Blood flow, Computational fluid dynamics, Biomedical engineering, Computational models, Cardiovascular biology, Interventional cardiology, Cardiovascular diseases

## Abstract

Obstruction of vessels is one of the most common health issues in the world and various methods of treatments have been offered, including medication therapy, stenting, and bypass surgery. However, recurrence of blockage is a major issue facing patients. The present work assesses the performance of a new innovative design of stents featuring two spirals, called ‘inserts’, which will create a rotating flow inside the vessel. Three different cases of artery and bypass configurations were studied and the results showed that the stent and inserts increased the axial and secondary velocity significantly. At a plane located 1 mm after anastomosis and at t = 0.25 s, the axial velocity was 23% higher for a case with stent and insert compared to the baseline configuration i.e., without stent/insert. In addition, it was found that the presence of the insert increases the wall shear stress (WSS). The maximum WSS among the three cases reached 113 Pa, which was 94% higher than the baseline case. The findings highlight the potential of the proposed stent/insert design in improving the hemodynamic parameters in the graft, anastomosis region, and the host artery, which in turn, could enhance the patency and longevity of the bypass graft.

**Supplementary Information:**

The online version contains supplementary material available at 10.1038/s41598-025-09613-8.

## Introduction

Biomaterials are widely used in medical applications, such as treating cardiovascular diseases. These materials, whether natural or synthetic, are designed in a way that allows them to integrate into living tissues^1^. One of the applications of biomaterials is the fabrication of stents and their placement in blood vessels. Stents are small, hollow tubes that hold open blocked or narrowed passageways, such as blood vessels. They are used to improve blood flow and alleviate blockages in medical procedures^2^. Sometimes blood vessels narrow for various reasons, such as the deposition of thin plaques^1^. For the treatment of vascular stenosis, such as coronary artery stenosis, several treatment options exist, including medical therapy, stent placement, and bypass surgery^3^. The choice of treatment depends on various factors, such as the severity and location of the stenosis, the overall health of patients, and the presence of other medical conditions. Due to the high significance of treating vascular occlusive diseases, researchers conducted numerous studies on coronary artery bypass grafts (CABG) and stent placements. Some of these studies were examined in the following paragraphs.

Kabinejadian et al.^4^ conducted a laboratory investigation to measure the velocity and shear stress in a newly graft-designed model of sequential anastomotic. This study investigated three models, including sequential anastomoses, conventional end-to-side anastomoses, and side-to-side anastomoses, under pulsatile flow conditions using particle image velocimetry (PIV) for velocity measurement. Finally, the researchers found that the new design, which is a combination of end-to-side and side-to-side configurations, outperformed the other models and showed better results. Kabinejadian et al.^5^ conducted a study to investigate and evaluate the numerical simulation of new helical/spiral anastomotic configurations for distal graft anastomoses. Finally, the researchers found that the helical configuration caused the generation of rotational flow, leading to increased wall shear stress (WSS) and reduced flow stagnation in the anastomotic region and host artery. Ruiz-Soler et al.^6^ utilized computational fluid dynamics (CFD) to investigate and optimize a spiral-induced bypass graft anastomosis. They found that the trailing edge orientation was the most crucial parameter in creating a rotational flow. Keshmiri et al.^7^ studied a numerical investigation on the impact of the geometry of a new design for bypass surgery. In the end, the researchers understood that the trailing edge orientation of 270 degrees exhibited better performance compared to 90 degrees in terms of generating strong rotational flow inside the anastomosis and the host artery. Liu et al.^8^ investigated a conical helical-inspired anastomosis graft to enhance rotational flow. In the end, the results demonstrated that the improved helical flow generated by the conical-helical-inspired graft leads to significantly high WSS. This action reduces the likelihood of thrombosis formation in the graft and consequently decreases the risk of graft occlusion. Frauenfelder et al.^9^ investigated the WSS of end-to-side and side-to-side anastomoses in coronary artery bypass grafting. Finally, they revealed that the greatest and smallest changes in wall shear stress occurred in the heel and the wall of the anastomosis, respectively. Furthermore, the side-to-side configuration had the highest level of WSS. Aboul-Hassan et al.^10^ conducted a study to investigate and compare the long-term effects of total arterial grafting with multi-arterial plus saphenous vein grafting. At the end of this study, the researchers found that patients who received multiple arterial grafts in addition to the saphenous vein graft had comparable long-term survival and freedom from adverse cardiovascular and cerebrovascular events compared to those who received complete arterial grafts. Stefil et al.^11^ investigated coronary artery bypass grafting in patients with diabetes and obesity. Finally, they understood that using a single internal thoracic artery resulted in fewer complications compared to using bilateral internal thoracic arteries. Tian et al.^12^ researched the relationship between Quantitative flow rates (QFR) and coronary artery bypass grafting outcomes. They found no significant correlation between the low flow rate ratio and the occurrence of vein graft occlusion.

Zhang et al.^13^ studied the fluid-solid interaction of stents-graft inside blood vessels. In the end, the researchers found that increasing the diameter of the blood vessel or reducing the length of the stent increases the von Mises stress applied to the blood vessel wall. Also determined that there is an inverse relationship between stress and stent stiffness. Cheng et al.^14^ investigated in-stent restenosis using finite element simulation. The results revealed that plaque growth and arterial wall behavior over time lead to a more uniform distribution of von Mises stress. Additionally, it was observed that tissue growth near the inner wall was more significant than in the outer layer. Antonini et al.^15^ validated a computational model for coronary stents. Finally, researchers understood that their free expansion simulation stents were sufficiently useful for validating the coronary stent model. Lin et al.^16^ investigated the effects of a self-expandable nitinol stent with a helical design. The results demonstrated that SmartFlex stents are safe in patients with femoral and popliteal artery disease. Martin et al.^17^ used CFD to investigate the impact of coronary stents and changes in vascular geometry. After performing simulations, the researchers observed significant differences in the distribution of WSS, time-averaged wall shear stress gradient (TAWSSG), and oscillatory shear index (OSI). Yu et al.^18^ conducted a study to compare and analyze the performance of conical and cylindrical stents in coronary arteries. The results demonstrated that cylindrical and conical stent placements improved hemodynamic parameters such as WSS and blood flow velocity distribution. Chen et al.^19^ investigated the commonly used curved stents in coronary arteries using CFD. Finally, the researchers found that the stent thickness is one of the most influential and critical factors affecting the WSS. Moreover, it determined that stents with a helical configuration create an additional swirling flow in the bloodstream. Wang et al.^20^ performed local hemodynamic analysis after stent implantation based on the Euler-Lagrange approach. Finally, the results showed that non-Newtonian blood models provide a more realistic description of blood flow and are more suitable for more accurate simulations.

Martin and Boyle^21^ conducted a sequential structural and fluid dynamics analysis of expandable coronary stents with balloon inflation. Finally, the results showed that the strut thickness is critical in designing coronary stents. Jayendiran et al.^22^ investigated stent grafting for endovascular repair of aortic aneurysms using fluid-solid interaction analysis. The researchers found that the material of the stent and the thickness of the stent struts significantly influence the mechanical behavior of the structure. Beier et al.^23^ conducted a study on the hemodynamics of stented arteries. They found that the distance between narrow struts in the stent can create larger regions with lower and higher WSS. However, these effects diminish as the size of the struts decreases. Razavi et al.^24^ used CFD to investigate the hemodynamics of stents in the main branch of coronary arteries. They determined that using a one-piece stent is better than a two-piece one. Dottori et al.^25^ investigated the mechanical behavior of peripheral stents and the interaction between stents and blood vessels. The researchers obtained results from their calculations for the self-expanding commercial stent, indicating that the radial force applied during deployment on the vessel wall may not be sufficient for the required degree of stenosis. Antonini et al.^26^ investigated the structural analysis of coronary stent implantation using finite element simulations. This study reviewed several studies and found that although the studies conducted in fields such as screening, industry, and employee training are credible, unresolved issues still require further investigation and research. Ibrahim et al.^27^ conducted a study to investigate the mechanical performance characteristics of ultra-thin coronary stents. In this study, two stents with a novel design were used, and they found that the first stent requires less bending force than the second stent. Furthermore, design 1 exhibited higher radial strength, less recoil phenomenon, and smaller shortening. Akhtar et al.^28^ investigated the computational fluid dynamics of stent placement within the artery. Finally, they found high blood pressure generated during stent placement inside the artery. After the stent placement, the velocity lines and normal pressure were restored.

Based on the literature, bypass surgery is among the last resort doctors perform for patients because this method requires open surgery. However, since this approach holds significant importance, extensive research was carried out to increase the lifespan of bypass grafts^4–12^. Moreover, it was determined that CFD had been used to perform numerous simulations on various types of stents and bypass grafts. The primary objective of most of these simulations was to investigate the WSS of the stent placement site^5,8,9,17–19,23^.

The innovation in the current study involves the effect of combining a stent and insert, which consists of two spiral loops and is placed inside the stent. Unlike previous studies, which typically simulated stents within simple arteries, this research places a stent within a bypassed artery for the first time. The primary goal of this research was to assess how combining stents with inserts could enhance stent performance under more complicated conditions.

In the following sections, the method used in the research will be described and its results will examine the effects of this combination on the performance of stents and blood flow in bypassed arteries.

## Methods

**Fig. 1:**
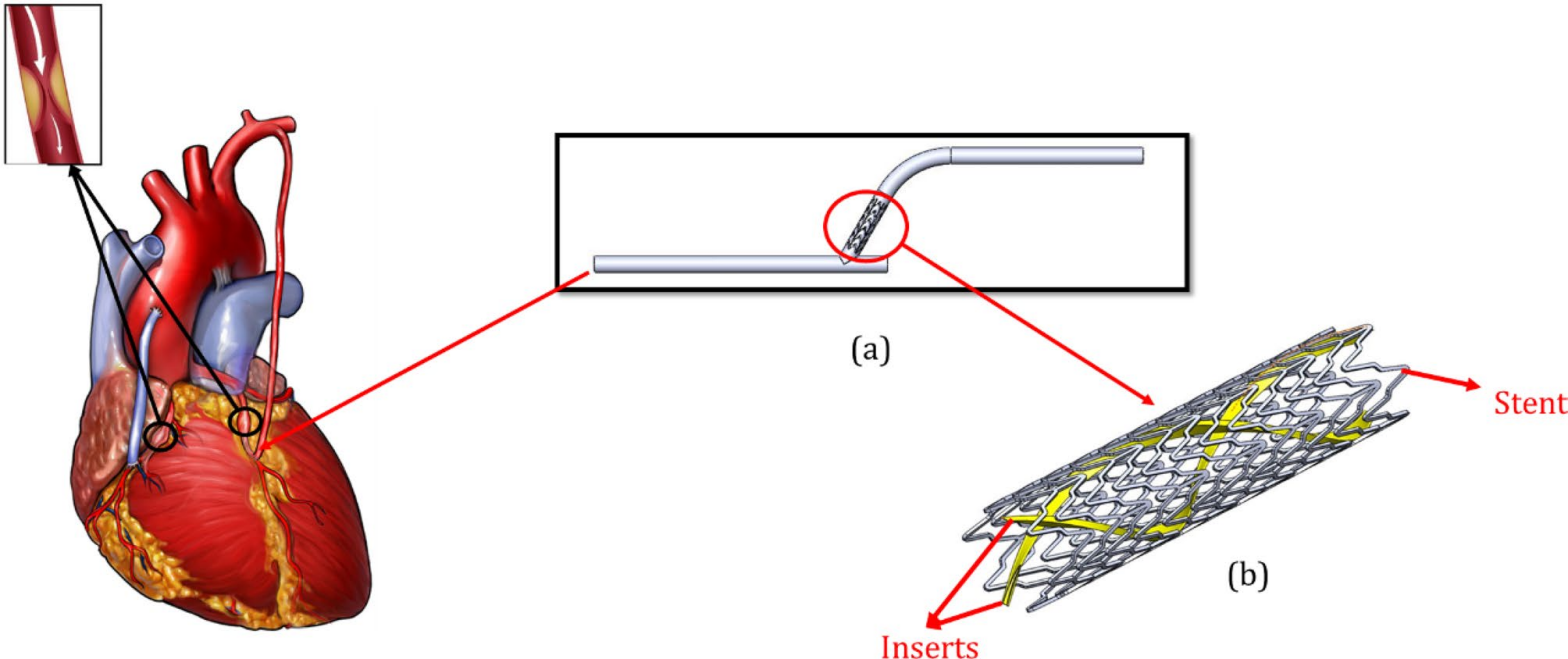
(**a**) The location of the stent in the bypass vessel and (**b**) the design of the new proposed stent and inserts configuration. This figure was created from four different images. The (a) and (b) were created by the present authors from a CAD file in SolidWorks 2019 software. first and second images from the left are taken from [drpavankumar.in] and [valleyhealth.com]. All images were modified and combined using PowerPoint 2021 software.

**Fig. 2:**
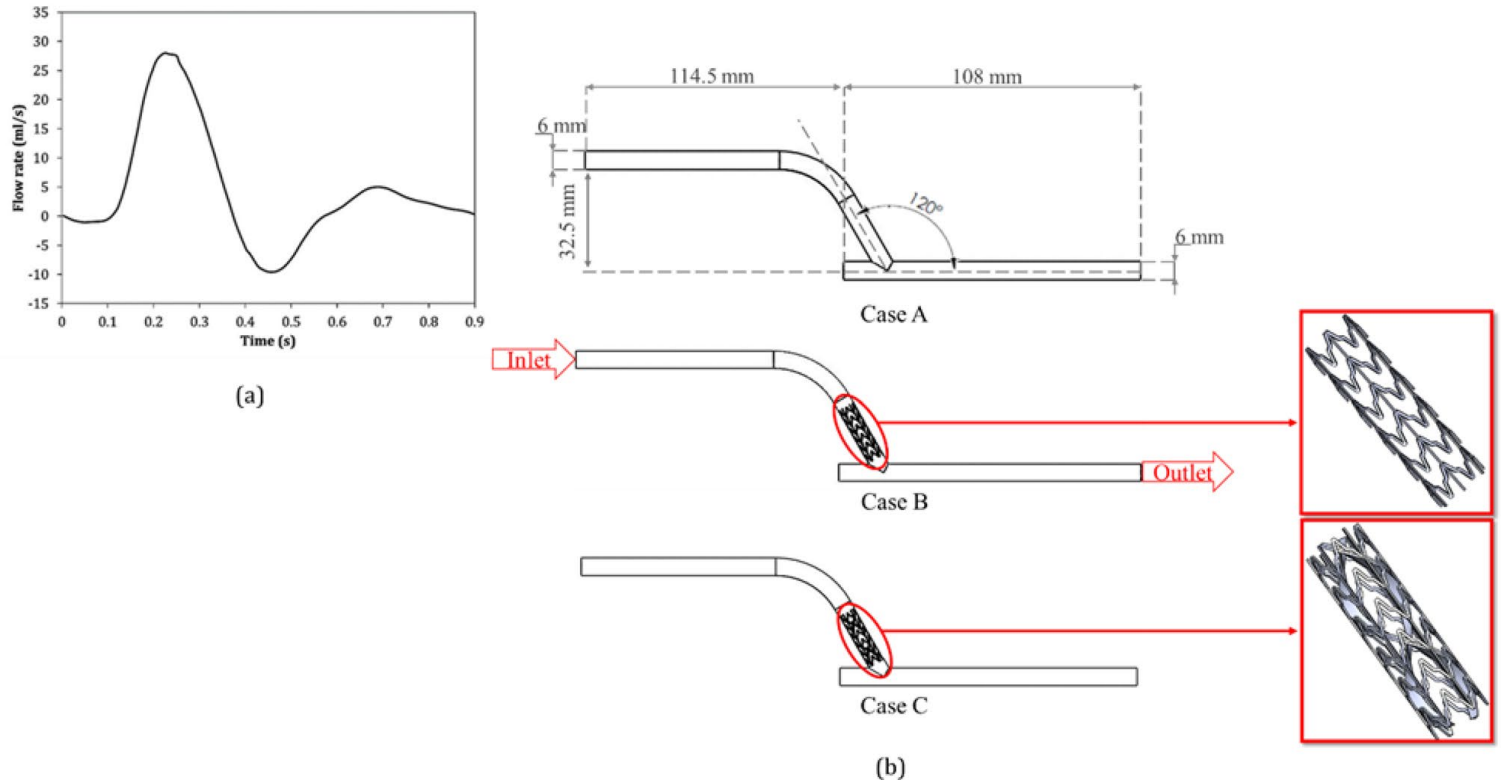
(**a**) The waveform of the inlet flow rate^5^, (**b**) three cases simulated in the present study (Case A: bypass without stent and inserts; Case B: bypass with stent; and Case C: bypass with stent and inserts).

In this study, a new innovative configuration is proposed, comprising a stent placed in a bypass vessel, as demonstrated in Fig. 1(a). The stent with insert is shown in Fig. 1(b). In order to investigate the effect of the insert, the waveform of the inlet flow rate is shown in Fig. 2(a). Three different configurations were then investigated, which are shown in Fig. 2(b).

The continuity and momentum equations (Eqs. 1–4) were solved using the Finite Volume Method (FVM) and the Semi-Implicit Method for Pressure-Linked Equations (SIMPLE) algorithm^29^.

1 $$\:\frac{\partial\:\rho\:}{\partial\:t}+\nabla\:.\left(\rho\:V\right)=0$$

2 $$\:\rho\:\left(\frac{\partial\:u}{\partial\:t}+u\frac{\partial\:u}{\partial\:x}+v\frac{\partial\:u}{\partial\:y}+w\frac{\partial\:u}{\partial\:z}\right)=-\frac{\partial\:p}{\partial\:x}+\mu\:\left(\frac{\partial\:{\tau\:}_{xx}}{\partial\:x}+\frac{\partial\:{\tau\:}_{yx}}{\partial\:y}+\frac{\partial\:{\tau\:}_{zx}}{\partial\:z}\right)+\rho\:{g}_{x}$$

3 $$\:\rho\:\left(\frac{\partial\:v}{\partial\:t}+u\frac{\partial\:v}{\partial\:x}+v\frac{\partial\:v}{\partial\:y}+w\frac{\partial\:v}{\partial\:z}\right)=-\frac{\partial\:p}{\partial\:y}+\mu\:\left(\frac{\partial\:{\tau\:}_{xy}}{\partial\:x}+\frac{\partial\:{\tau\:}_{yy}}{\partial\:y}+\frac{\partial\:{\tau\:}_{zy}}{\partial\:z}\right)+\rho\:{g}_{y}$$

4 $$\:\rho\:\left(\frac{\partial\:w}{\partial\:t}+u\frac{\partial\:w}{\partial\:x}+v\frac{\partial\:w}{\partial\:y}+w\frac{\partial\:w}{\partial\:z}\right)=-\frac{\partial\:p}{\partial\:z}+\mu\:\left(\frac{\partial\:{\tau\:}_{xz}}{\partial\:x}+\frac{\partial\:{\tau\:}_{yz}}{\partial\:y}+\frac{\partial\:{\tau\:}_{zz}}{\partial\:z}\right)+\rho\:{g}_{z}$$

τ is the shear stress in Eqs. (2–4). The Carreau viscosity model was chosen for this study in order to account for non-Newtonian effects^30–35^. The connection for the shear stress of the Carreau viscosity model was represented by Eqs. (5–8)^36,37^.

5 $$\:\tau\:=\mu\:D$$

6 $$\:D=\frac{1}{2}\:[grad\:V-(grad\:V{)}^{T}]$$

7 $$\:\gamma\:=\sqrt{2tr\left({D}^{2}\right)}$$

8 $$\:\mu\:={\mu\:}_{\infty\:}+\left({\mu\:}_{0}-{\mu\:}_{\infty\:}\right)\left[\right(1+({\Gamma\:}\gamma\:{)}^{2}{\left)\right]}^{\frac{n-1}{2}}$$

In Eq. (8), $\:{\mu\:}_{0}$ is zero shear rate with amount of 0.022 ($\:\frac{kg}{ms}$) and $\:{\mu\:}_{\infty\:}$ is infinite shear rate with amount of 0.0022 ($\:\frac{kg}{ms}$). Inaddition, $\:{\Gamma\:}$ show the time constant and *n* indicates power-law index. For blood, the density, the time constant, and power-low index are 1050 ($\:\frac{kg}{{m}^{3}}$), 0.11 (*s*), and 0.392, respectively^16^. The maximum Reynolds and Womersley numbers, based on the standard Newtonian blood viscosity value of 0.0035 (Pa.s), are determined to be Re_max_ = 1776 and α = 4.34, respectively, and the peak flow rate is Q_max_ = 27.9 $\:\left(\frac{ml}{s}\right)$^5^.

The Carreau viscosity model was chosen for this study in order to account for non-Newtonian effects. At the graft inlet, a fully developed pulsatile flow was applied, and at the artery outlet, the traction-free outflow boundary condition was used. All walls were subject to a no-slip boundary constraint, and a rigid wall model was presumed.

This study used an idealized straight-cylinder model of the bypass graft to investigate the impact of the insert design on local hemodynamics. In stent/hemodynamics research, straight-tube models are a well-established first step that allows the unambiguous attribution of observed flow variations to device characteristics rather than anatomical variability. Moreover, this assumption enables systematic comparison and mesh-independence testing, but it always excludes curvature-induced secondary flows and the corresponding distortion of velocity profiles. LaDisa et al.^38^ demonstrated that when a naturally curved coronary artery was straightened, the time-averaged WSS distributions changed by 15–25% compared to the curved shape. Moreover, The Left Anterior Descending (LAD) artery is one of the primary vessels prone to occlusion, and owing to its relatively uniform geometry and gentle curvature, it can be approximated as a straight conduit in simulations without significantly affecting the accuracy of hemodynamic predictions.

The present stent-insert geometry is a new one and it is considered a constant diameter artery. The real coronary arteries taper from proximal to distal and other researches shows a reasonable assumption to consider a constant diameter model. Liu et al.^39^ compared branch coronary vessels and curved coronary in equal-diameter and conical expansions. They found that peak WSS differed by only 3.8% in branch vessels (75.21 Pa vs. 72.45 Pa) and 7.2% in curved vessels (39.43 Pa vs. 36.56 Pa) using a conical rather than uniform diameter assumption. These findings demonstrate that hemodynamic trends can be captured by straight-tube approximations with an error of less than 8%. Fogell et al.^40^ demonstrated that incorporating arterial elasticity via FSI reduced median time-averaged wall shear stress (TAWSS) by 5.7% compared to rigid‐wall CFD. Accordingly, the rigid‐wall model provides a conservative way to investigate the hemodynamic effects of the intraluminal insert design.

Ansys Fluent (Version 2020 R2) was used for these simulations. A SIMPLE scheme was used for simulations. Moreover, the spatial discretization for gradient, pressure, and momentum was least squares-based, second-order, and second-order upwind, respectively. Second-order implicit was used for the transient formulation. To maintain numerical stability in an unstable CFD simulations, the fluid must not traverse more than one cell length in a single time step, a requirement known as the Courant–Friedrichs–Lewy (CFL) condition. The CFL number is shown by Eq. (9).

9 $$\:CFL=\frac{u\varDelta\:T}{\varDelta\:X}$$

where, u is the local flow velocity, $\:\varDelta\:T$ is the time step, and $\:\varDelta\:X$ is the smallest grid spacing.

The Oscillatory Shear Index (OSI) is hemodynamic parameter used in this investigation, were computed using Eq. (10)^41,42^.

10 $$\:OSI=0.5\left(1-\frac{\left|{\int\:}_{0}^{T}{\overrightarrow{\tau\:}}_{W}dt\right|}{{\int\:}_{0}^{T}\left|{\overrightarrow{\tau\:}}_{W}\right|dt}\right)$$

**Fig. 3:**
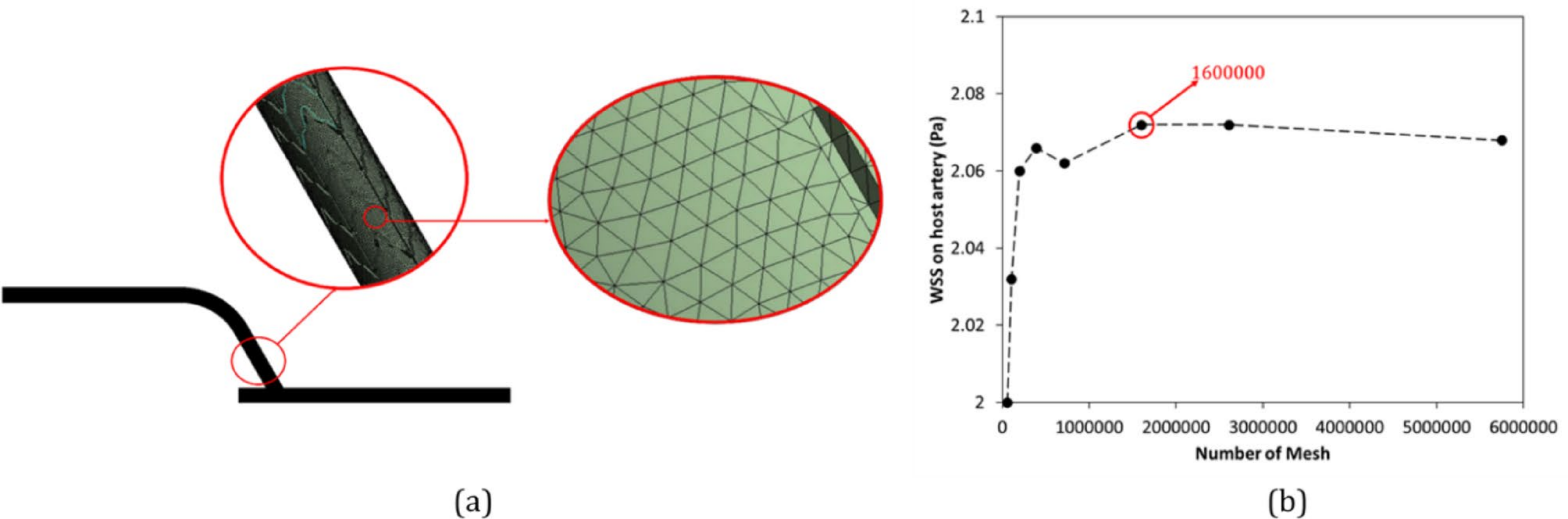
(**a**) The schematic image of the chosen mesh around the stent and insert, and (**b**) The mesh independency diagram for the parameter of WSS on host artery.

Figure 3(a) shows the mesh utilized in this simulation. Additionally, Fig. 3(b) depicts the mesh independence study, demonstrating that based on the WSS difference, the mesh with 1.6 m cell (size of each element was 0.2 mm) has sufficient resolution and is taken forward for all subsequent simulations.

## Validation

**Fig. 4:**
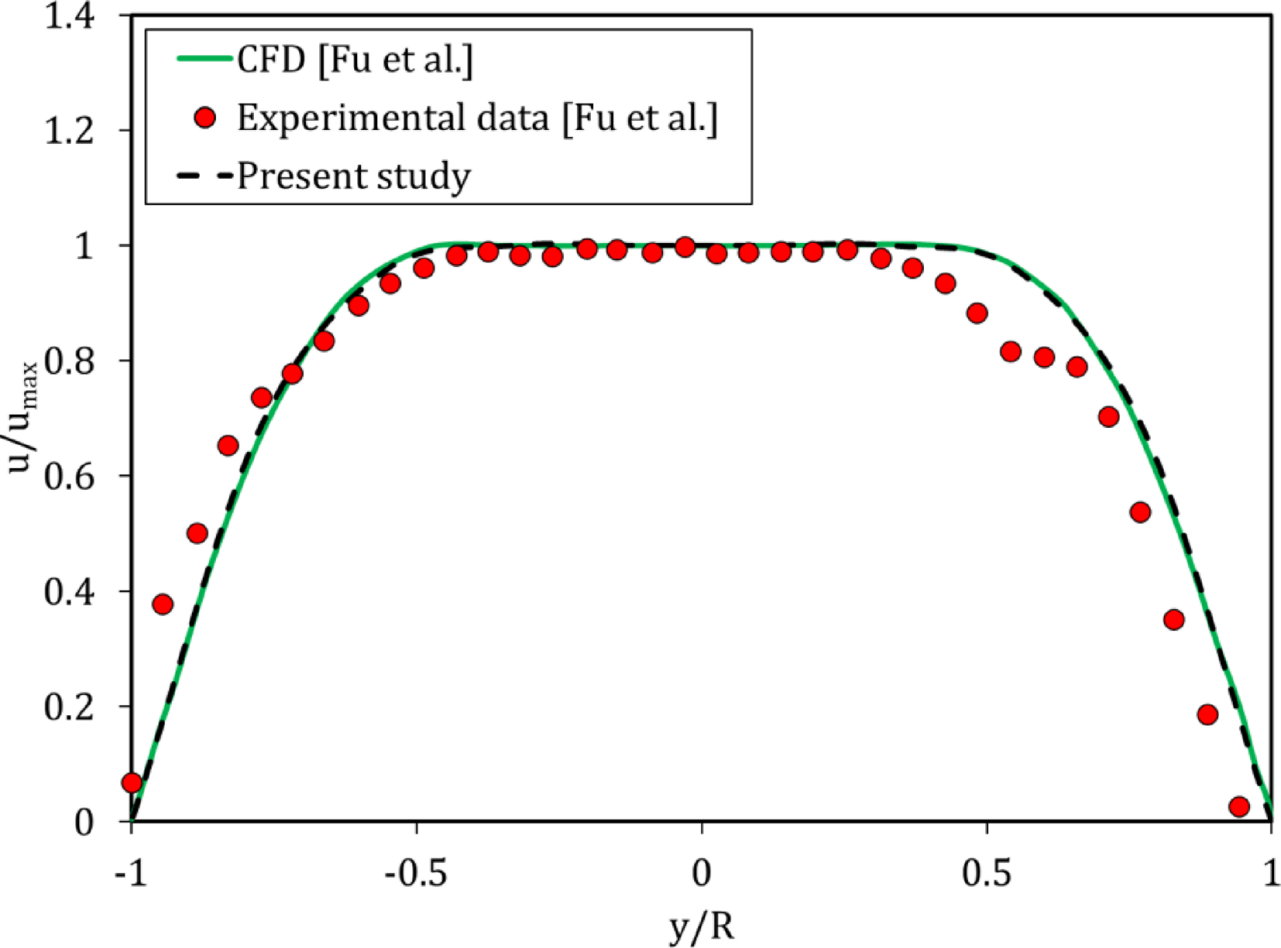
Normalized velocity profiles in the middle plane of the 600 μm×600 μm microchannel.

A microchannel was examined to ensure accuracy and correct operation in the simulation. The cross-section of this microchannel was 0.6 × 0.6 mm and its length was 7.2 mm. Reynolds number was considered 0.04. The simulated flow was PAAm 1.25% AN905SH solution and the parameters of the Carreau model are presented in Table 1. Finally, Fig. 4 shows the results of the present simulation compared against experimental tests and simulations of other researchers at the middle of the microchannel^43^. It was found that the simulation performed in this study was accurate.

**Table 1:** The characteristics of the Carreau model for 20 °C PAAm AN905SH solutions used in the study by Fu et al.^43^.

AN 905SH solutions	$\:{\eta\:}_{0}\:\left[Pa\:s\right]$	$\:{\eta\:}_{\infty\:}\:\left[Pa\:s\right]$	$\:\lambda\:\:\left[s\right]$	$\:n$	$\:\rho\:\:\left[\frac{kg}{{m}^{3}}\right]$
1.25%	52.51	0.064	18.93	0.27	1000

## Results and discussion

**Fig. 5:**
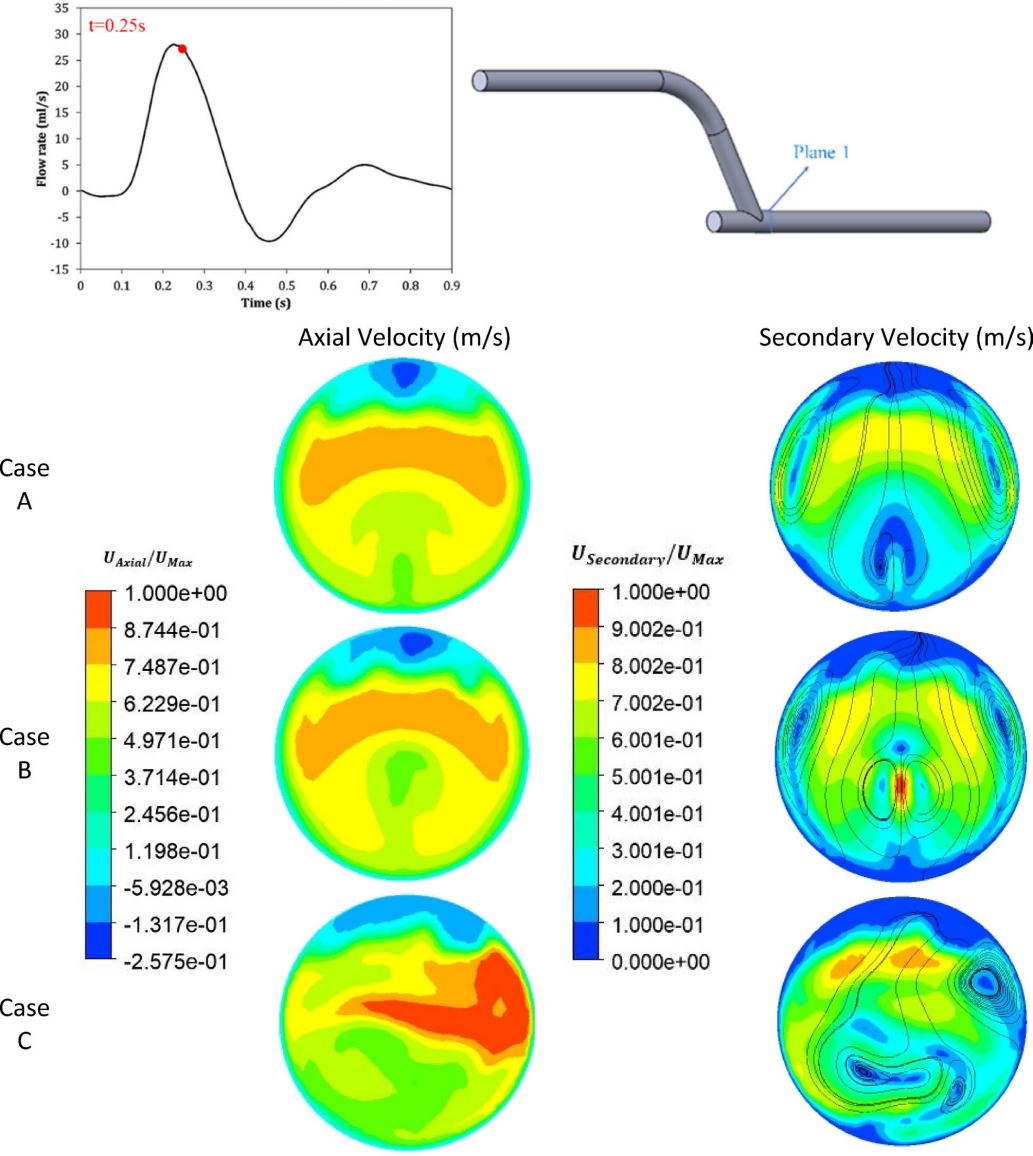
The comparison of the axial and secondary velocity at t = 0.25 s and at a distance of 1 mm from the bypass for all three cases.

**Fig. 6:**
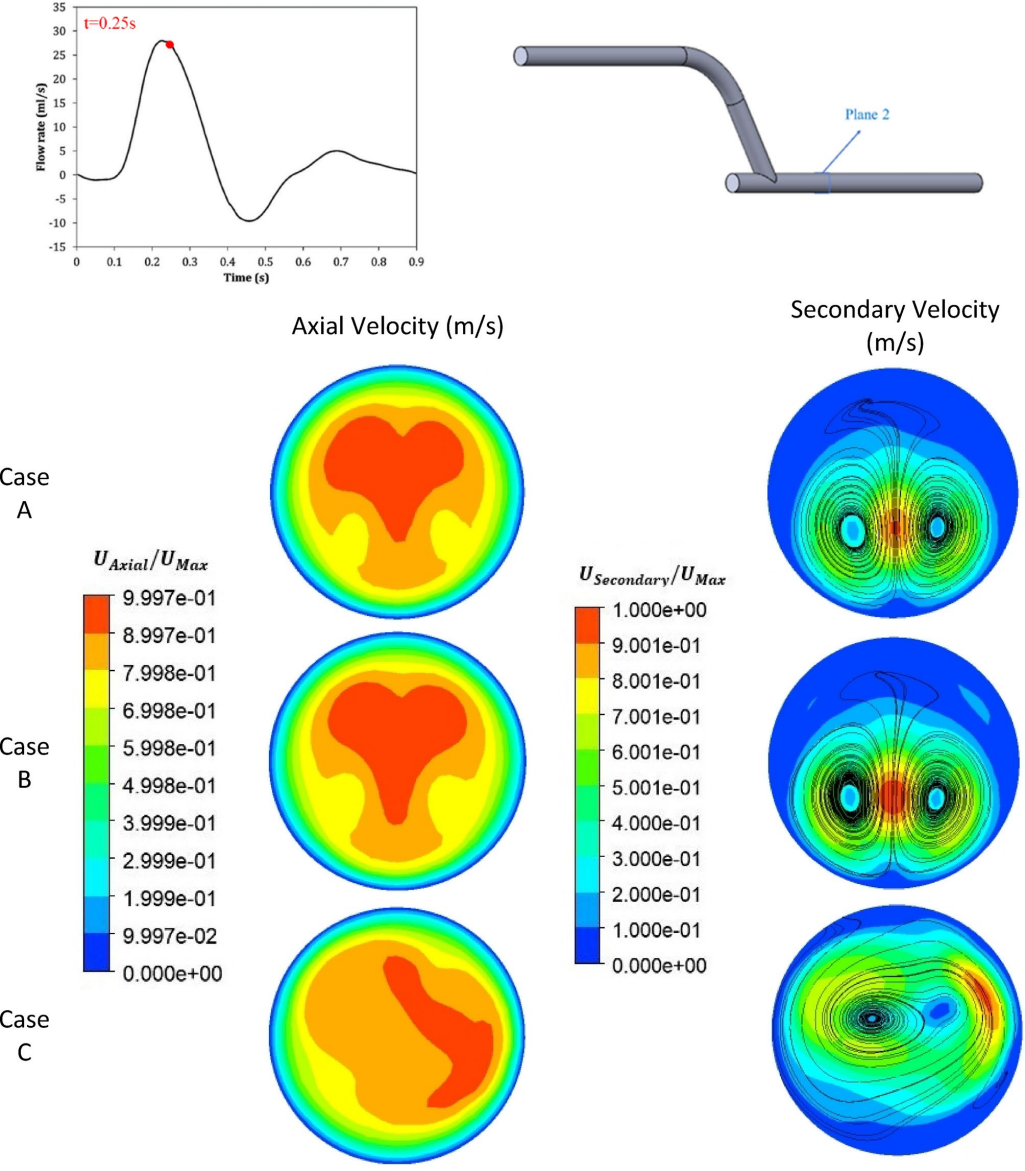
The comparison of the axial and secondary velocity at t = 0.25 s and at a distance of 50 mm from the bypass for all three cases.

**Fig. 7:**
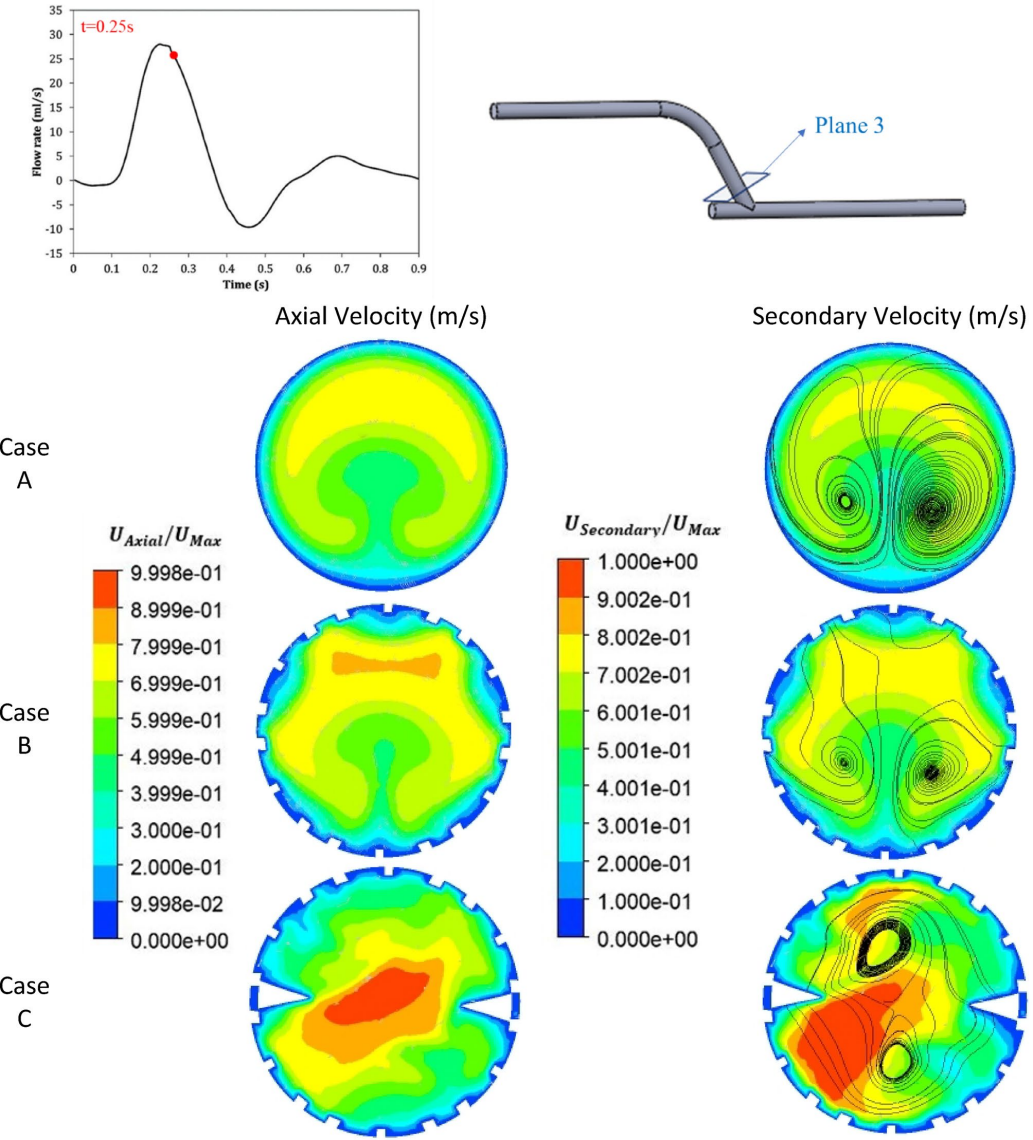
The comparison of the axial and secondary velocity at t = 0.25 s and at normal to bypass for all three cases.

The time step in the present simulations was set base on CFL ≤ 0.5 (corresponds to 0.05 s). The time step in the present simulations was set to 0.001 s to avoid numerical wiggles and data were recorded every 0.01 s. The simulations were carried out four times to eliminate the transient flow start-up effects, and the outcomes of the fourth period are displayed. Figures 5, 6 and 7 show the contours of axial velocity and secondary velocity in three cross-sections located at 1 mm, 50 mm from the anastomosis and normal to the graft at the anastomosis (i.e., ‘Plane 1’, ‘Plane 2’, and ‘Plane 3’, respectively) at 0.25 s (at systole).

Figure 5 shows the contours of the axial velocity and the secondary velocity at a distance of 1 mm from the bypass in all three cases of the studied geometry and at t = 0.25 s. As shown in this figure, the axial velocity in Case A was more uniform and had fewer sudden changes. Furthermore, the regions with low or high velocities were separated. In Case B, more changes in velocity distribution were observed, especially in the central areas where the speed decreased. However, most of the changes occurred in Case C, and it was evident that the insert increased the flow inhomogeneity and created high local velocities. In addition, according to the study of Kabinejadian et al.^5^, the presence of the spiral flow increased the axial velocity in parts of the artery and, as a result, increased the WSS, which is consistent with the results obtained in this study. Comparing Cases B and C showed that the addition of a protrusion (insert) caused changes in velocity and reduced low-velocity areas, which is consistent with the results of^6,44^.

The secondary velocity in Case A was symmetrical, as seen in Fig. 5. The flow pattern was between 0.26 and 0.65 m/s and weak vortices were observed. In Case B, the secondary velocity changed significantly and a high-velocity region was created in the center, indicating the effect of the stent. In Case C, the flow pattern became more complex, the low-velocity regions were reduced, and the stent with the insert increased the size and intensity of the vortices, which moved closer to the wall and increased the WSS. Overall, the insert increased the secondary velocity and intensity of the vortices and reduced stagnation areas^5–7,44^.

The axial and secondary velocity at distance of 50 mm from bypass and at time of 0.25 s were shown in Fig. 6. In this figure unlike Case C where the peak axial velocity was closer to the wall, in Cases A and B, the axial velocity patterns were symmetrical at the center of the plate and had the same value of 1.47 m/s.

In Fig. 6 the secondary and streamlines in Cases A and B show the flow was relatively symmetrical, and there were two dominant vortices at the center of the artery, but the intensity of the vortices in these cases were lower, and the streamlines were more uniform and distinct. Moreover, the maximum secondary velocity was about 0.22 m/s, and the concentration of velocity was higher in the center and near the vortices, but the secondary velocity around the walls was low in both cases, which can reduce the WSS and thus increased risk of stenosis; this behavior was also observed in the study of Kabinejadian et al.^5^ and Keshmiri et al.^7^.

In Case C, the presence of the insert changed the flow pattern, and unlike Cases A and B, the flow becomes completely asymmetrical. Similarly, the secondary velocity pattern changes completely, replacing the two-vortex pattern with an asymmetrical single dominant vortex, highlighting the effectiveness of the insert in creating a spiral flow in the artery. According to some of the previous studies, the proximately of the secondary flow streamlines to the side wall, tends to improve the hemodynamic parameters^5–7,44^.

The axial and secondary velocities at the normal to bypass and at t = 0.25 s (at systole) were shown in Fig. 7. In Cases A and B, the axial velocity contour shows symmetrical pattern but the maximum velocity in Case B was 13% ​​higher than in Case A, which was due to the presence of the stent and the smaller cross-sectional area of ​​the passageway. This was visible in Case C, which also had a stent insert and the creation of a rotating flow, increasing axial velocity by 25% and 11% compared to Cases A and B, respectively, which was consistent with the results of^5–7,44^.

The secondary velocity and streamlines in Fig. 7 show that, in Cases A and B, the secondary flow was symmetrical and relatively uniform; two large and regular vortices were observed that were symmetrically located on both sides of the center of the contour. Streamlines were relative smooth without any abrupt changes, consistent with the observations of^5,7^. In Case C, however, the high-velocity region was expanded compared to the previous two cases, creating a strong asymmetrical pattern. Moreover, the low-velocity regions were reduced in size, which could be an important factor in delaying artery occlusion^5–7,44^.

The results at t = 0.41 s in the above three monitoring planes are presented in the appendix (see Supplementary Figure S1 to S3 online). In these figures, one could see that adding the insert leads to strong vortices forming closer to the vessel wall, increasing the velocity near the walls, which in turn would increase the WSS, with potential benefits on the hemodynamics in the artery.

Tables 2 and 3 were shown Comparison of axial velocity and secondary velocity at t = 0.25 s. Based on Table 2 at 1 mm after anastomosis, Case C exhibits the highest peak axial velocity at 1.84 m/s, in contrast to 1.56 m/s for Case B and 1.50 m/s for Case A. Moreover, at 50 mm after anastomosis, all cases converge toward the baseline. However, Case C has a higher maximum still (1.47 m/s compared to 1.46 m/s for Case A) that reflects continued augmentation of forward flow, and at a plane normal to bypass, Case C has the peak value of 1.90 m/s, which reflects most momentum gain by the host vessel. Furthermore, the mean axial velocity remains approximately constant (1.01 m/s) across all scenarios and planes, proving the total flow rate retention.

Table 3 shows the secondary velocity at t = 0.25 s, at 1 mm after anastomosis. Case C yields a strong peak secondary flow (1.15 m/s), only slightly below Case B (1.30 m/s) but above Case A (1.05 m/s), reflecting robust local mixing. In the 50 mm position after anastomosis, Case C secondary peaks of 0.22 m/s are consistent with those of Cases A and B, showing that the heightened perturbation dissipates over time without creating downstream oscillations. At a plane normal to bypass, Case C has the most significant secondary peak value (1.74 m/s) and the most considerable average value (1.03 m/s) and is thus able to drive cross-stream mixing into the host artery better than either Case A or Case B.

**Table 2:** Comparison of axial velocity in different modes at t = 0.25 s.

Plans		Min Axial Velocity (m/s)	Max Axial Velocity (m/s)	Average Axial Velocity (m/s)
1 mm after anastomosis	Case A	-0.47	1.50	1.01
	Case B	-0.47	1.56	1.01
	Case C	-0.19	1.84	1.01
50 mm after anastomosis	Case A	0	1.46	1.01
	Case B	0	1.47	1.01
	Case C	0	1.47	1.01
Normal to bypass	Case A	0	1.43	1.01
	Case B	0	1.53	1.04
	Case C	0	1.90	1.07

**Table 3:** Comparison of secondary velocity in different modes at t = 0.25 s.

Position		Min Secondary Velocity (m/s)	Max Secondary Velocity (m/s)	Average Secondary Velocity (m/s)
1 mm after anastomosis	Case A	0	1.05	0.48
	Case B	0	1.30	0.53
	Case C	0	1.15	0.50
50 mm after anastomosis	Case A	0	0.21	0.05
	Case B	0	0.22	0.06
	Case C	0	0.22	0.07
Normal to bypass	Case A	0	1.23	0.88
	Case B	0	1.32	0.90
	Case C	0	1.74	1.03

Overall, Case C produces the highest peak systolic axial acceleration and cross-flow mixing at each point measured, without compromising mean flow rate or creating undue downstream perturbations. These quantitative results highlight Case C as the new design for promoting local hemodynamics at physiologic (systolic) flow rates. Li et al.^45^ showed that even moderate elevations in laminar wall shear stress promote endothelial quiescence, inhibit smooth-muscle proliferation, and reduce neointimal hyperplasia. These promising simulation results will guide the laying of the groundwork for eventual clinical application.

**Fig. 8:**
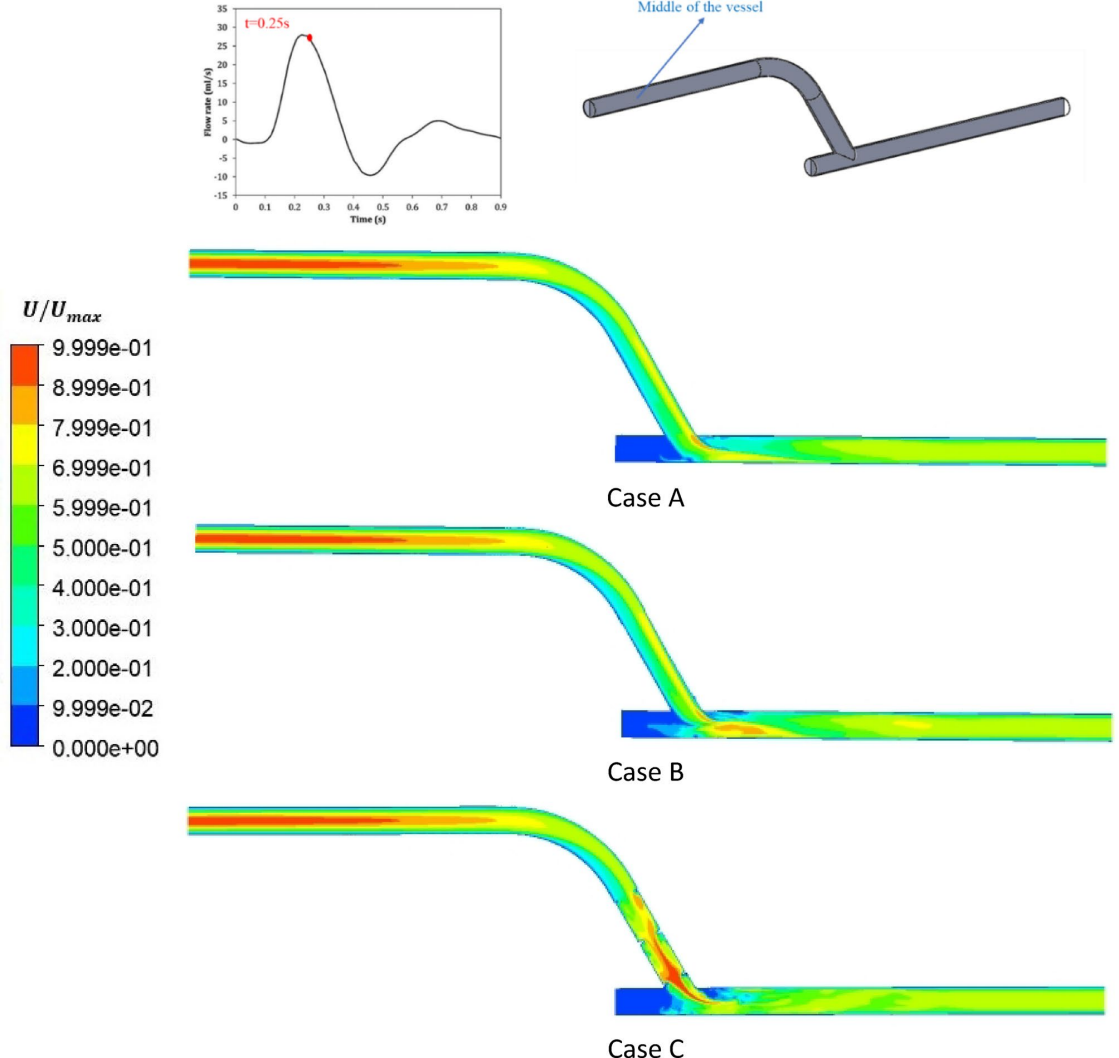
The velocity contour in the middle of the vessel at t = 0.25 s.

The velocity contours at the middle of three cases at t = 0.25 s (at systole) are shown in Figs. 8.

In Fig. 8, the impact of adding the stent and the insert on the velocity magnitude in the bypass graft, anastomosis and the host artery is evident, with Case C displaying significant changes in the flow pattern. In Case C, at t = 0.25 s, the high-velocity region was centrally located, but at diastole (at t = 0.41 s - see Supplementary Figure S4 online), the location of high- and low-velocity regions look very different, as one would expect.

**Fig. 9:**
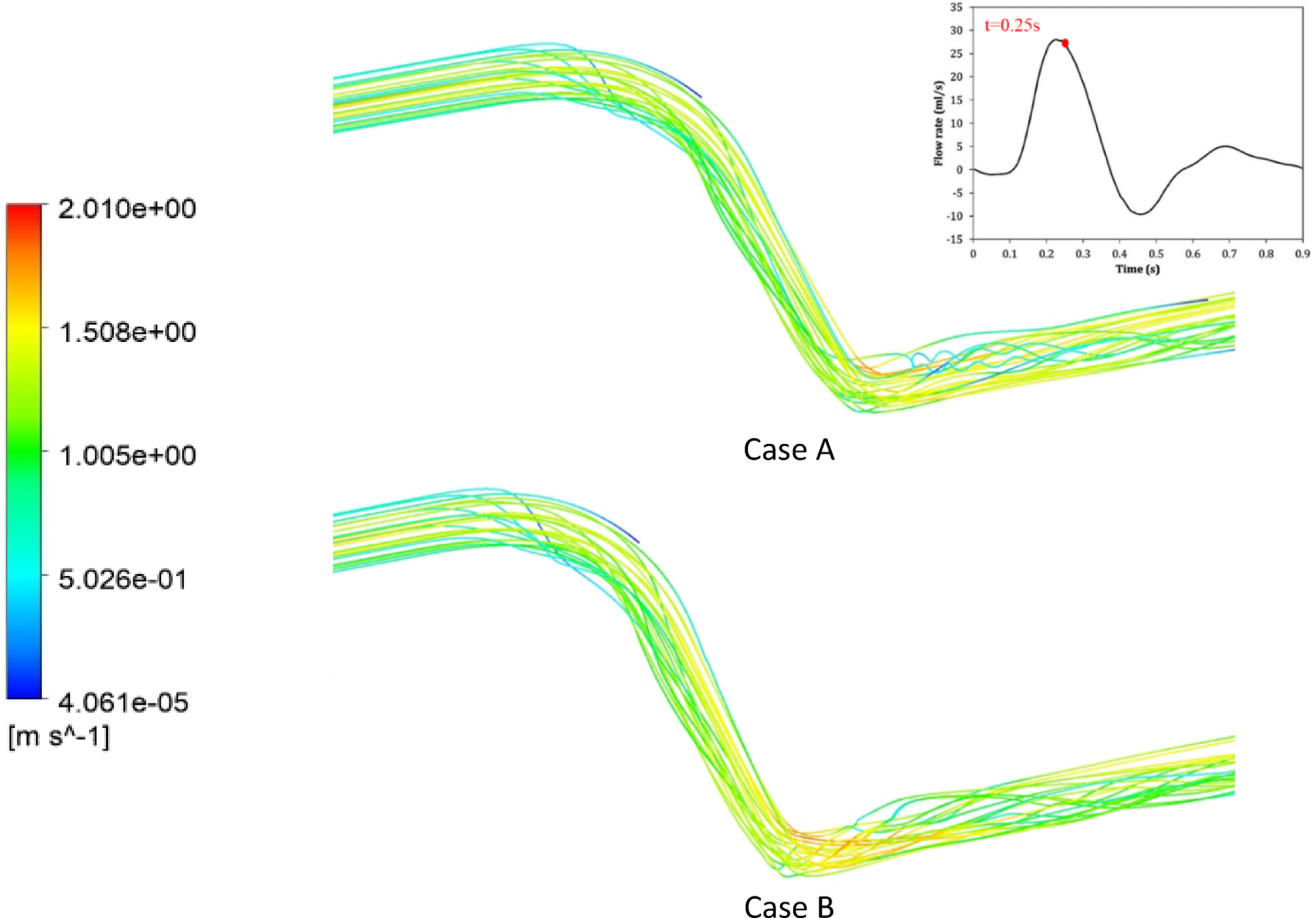

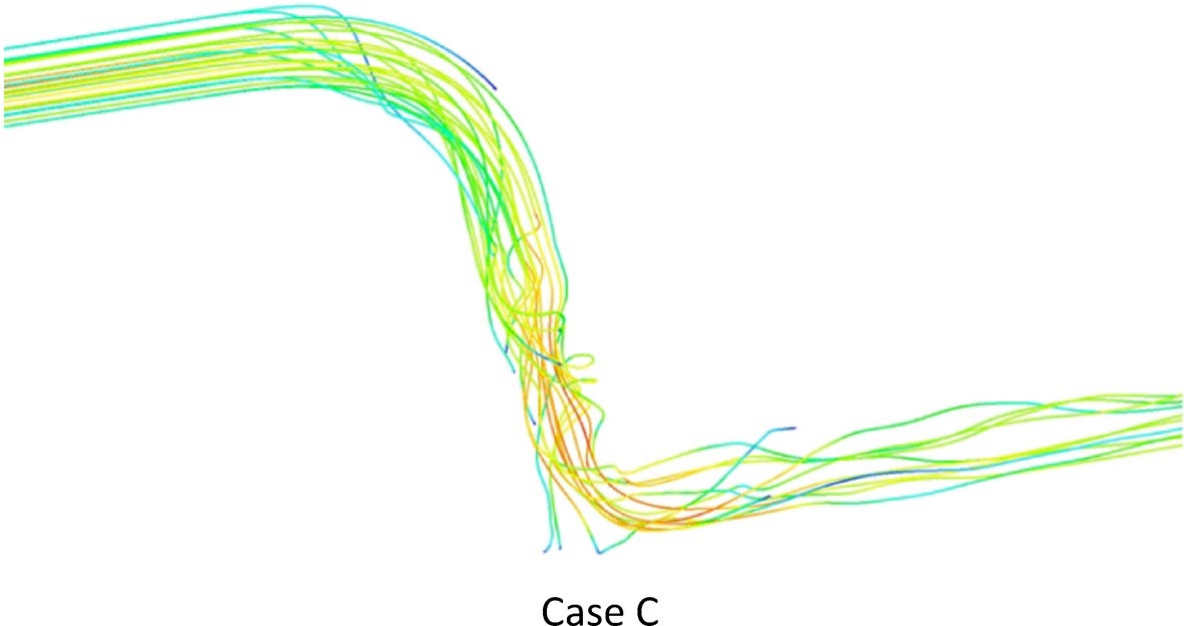
The streamlines velocity in the bypass area at t = 0.25 s.

The velocity streamlines at the around the anastomosis are shown in Figs. 9 for all three cases at systole. Cases A and B display streamlines that are uniform and not showing any significant separation, while in Case C, the insert leads to severe disturbances in the flow. In fact, the impact of the insert is even more pronounced at diastole (see Supplementary Figure S5 online).

**Fig. 10:**
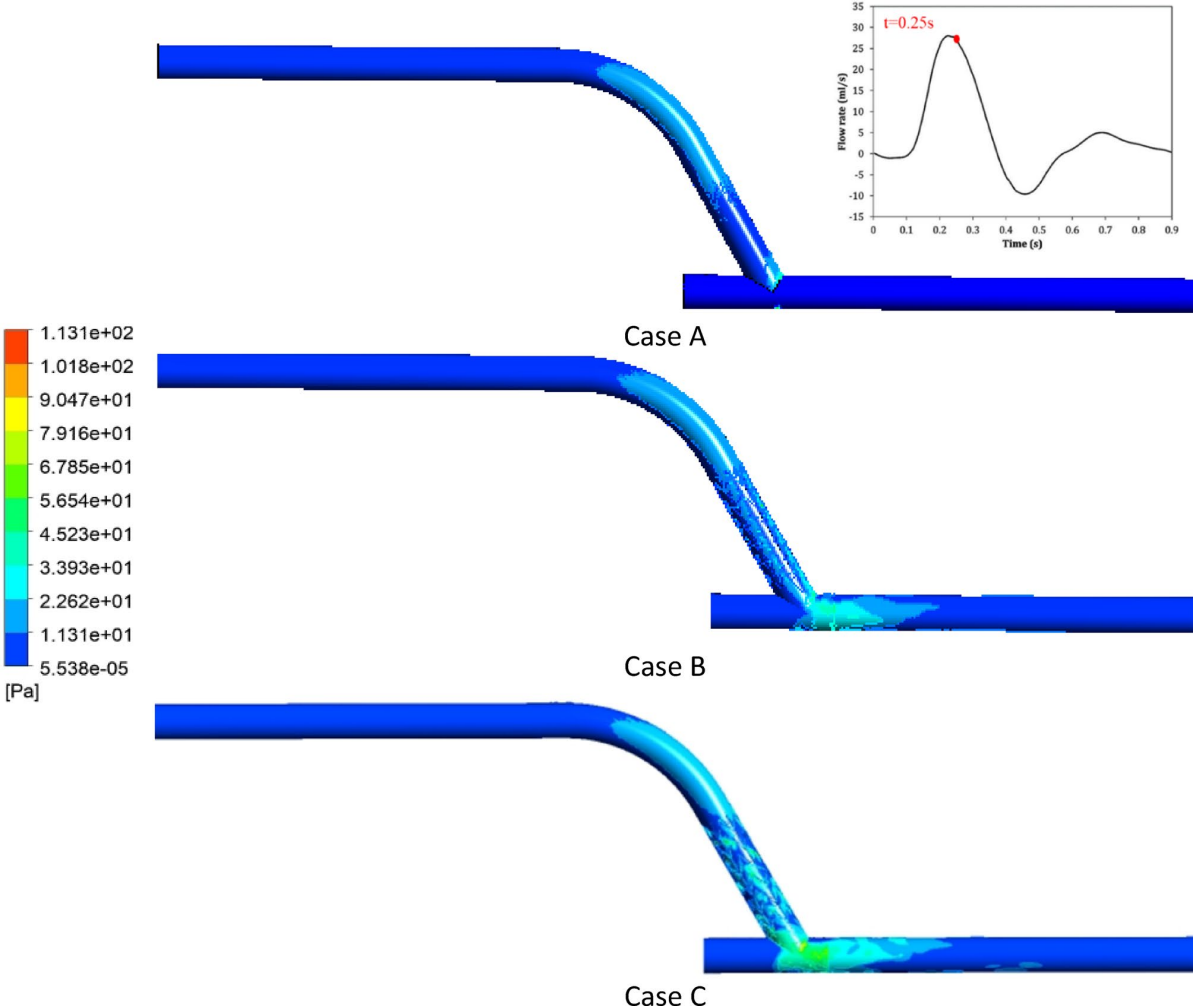
The WSS contours at t = 0.25 s.

The contours of the WSS in the three cases are shown in Fig. 10. WSS in Case A was stable and rather uniform, while adding the stent in Case B, led to the expansion of the high-WSS region, particularly around the stent and the anastomosis. In Case C, the elevated-WSS region near the anastomosis was extended even further compared to Case B, indicating the impact of the spiral flow on the local hemodynamics. Consistent with the observations from Figs. 8 and 9, under diastole (see Supplementary Figure S6 online), the difference between Case C and B is even more significant. in Fig. 10, it was also found that the WSS in the area where the stent struts were located was higher compared to the gaps in-between the struts. However, adding an insert further increases the WSS in the space between the stent struts, which is something deemed positive in terms of delaying any potential re-occlusion^46,47^. To prevent thrombus formation and neointimal hyperplasia, the insert design reduces low and oscillatory shear areas and increases near-wall WSS and continuous swirling flow. Chen et al.^48^ numerically demonstrated that a similar swirling flow increases mean WSS by 20%. Moreover, Chen et al.^49^ showed that monocytes (U-937 cells) adhered to a silicone tube that had circular-ring stents inserted. The induced swirling flow considerably reduced the adhesion density of U-937 cells to the surface in the disturbed flow zones to a level equivalent to that achieved when no stents were inserted into the tube. These results indicate that the hemodynamic modifications of the insert will likely reduce acute and chronic thrombotic risk.

**Fig. 11:**
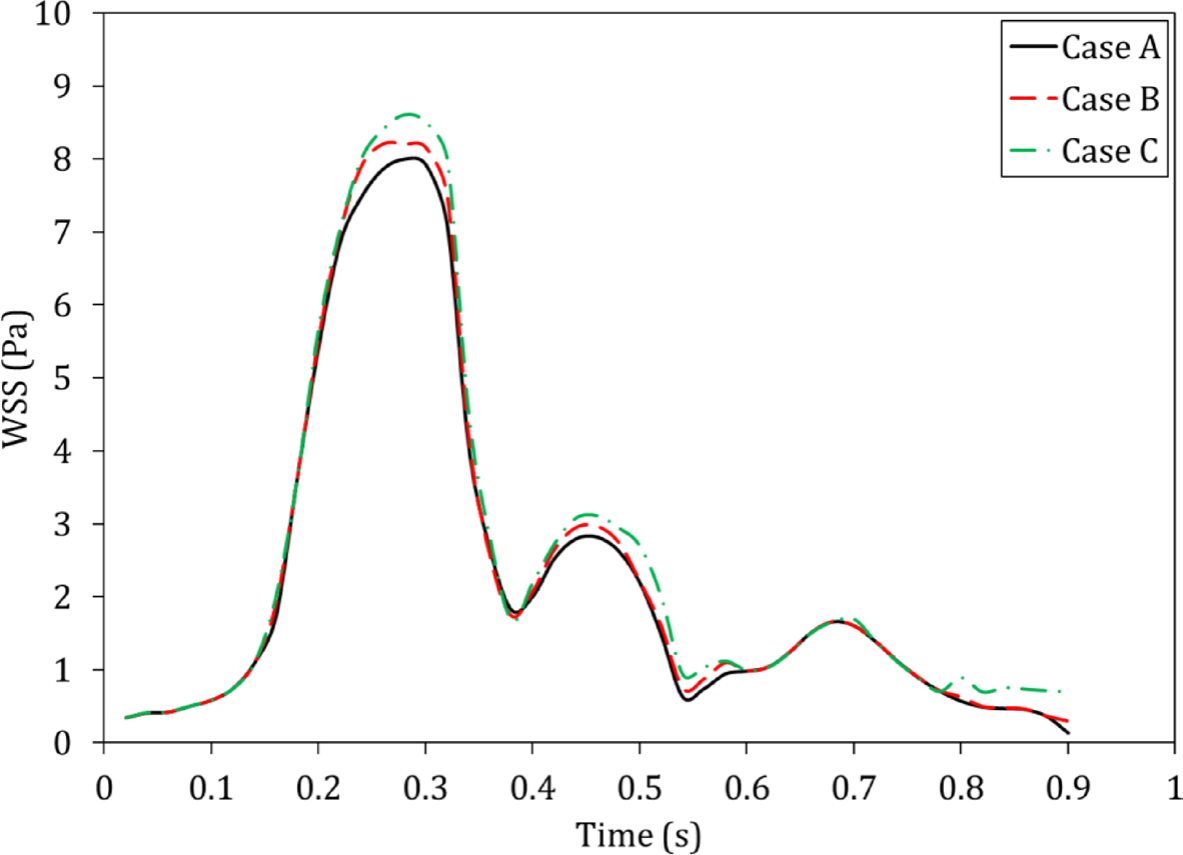
The average WSS over one full cardiac cycle on host vessel.

Figure 11 shows the average WSS over one full cardiac cycle on host vessel.

According to Fig. 11, the average WSS in Case C was higher than in Cases A and B. Moreover, at the critical average WSS (WSS < 0.5 Pa) was at t = 0.9s in Cases A and B, but in Case C, WSS at t = 0.9s was improved (0.7 Pa). Since risk for thrombus formation is highest when flow is lowest during diastole, elevated WSS throughout the cardiac cycle is necessary. The complete-cycle WSS profiles (Fig. 11) indicate that the insert (Case C) maintains shear above thrombosis-preventing thresholds even in diastole. Such persistent prevention of the low-shear states indicates that the insert can provide long-lasting anti-thrombogenic advantages in vivo. Furthermore, in case C, the mean WSS at t = 0.28 s increased by 12.67% compared to case A and 4.87% compared to case B, representing the most significant shear increase of the three designs. Importantly, mechanistic studies have shown that even slight elevations in the relaxed WSS can significantly inhibit endothelial cell proliferation and reocclusion^45^.

**Fig. 12:**
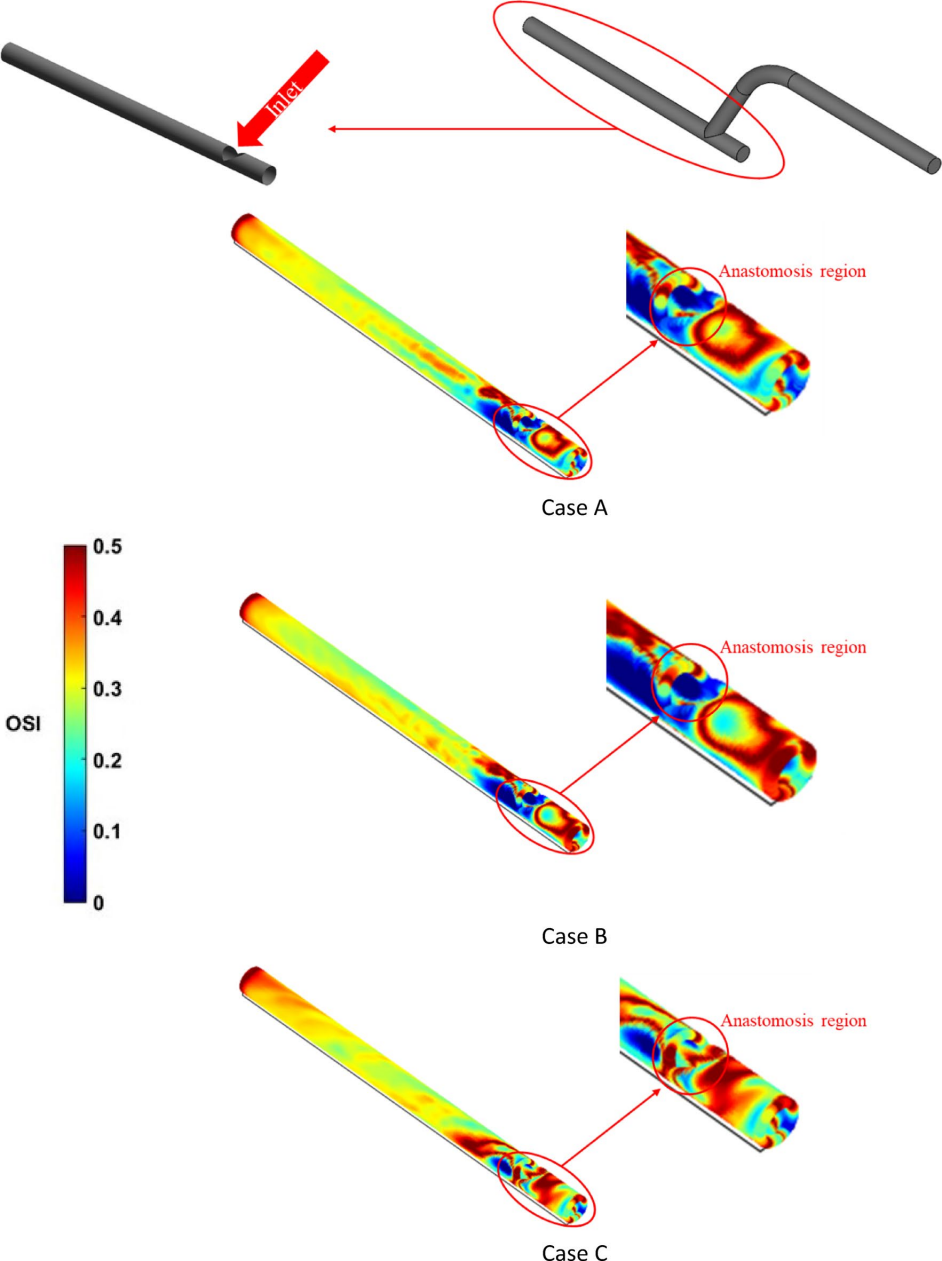
The OSI for the host vessel.

Figure 12 compares the OSI distribution for all three cases, showing that the anastomosis region towards the host artery had the highest OSI. Once again, Cases A and B exhibit similar patterns for OSI rate, while Case C shows a completely different OSI pattern, particularly in proximity of the anastomosis region, due to the spiral flow generated by the insert. In addition, distinct areas of high OSI in the occluded part of the host artery were generally associated with secondary flow fluctuations. This pattern indicates the secondary flow fluctuation caused by the rotation of the bulk flow at the anastomosis^5^.

## Conclusions

Three cases of bypass grafts were modeled in the present study to assess the impact of a novel insert concept. Three cases simulated here consisted of: Case A was the geometry of the host vessel and bypass without any stent, Case B was the host vessel and bypass with a stent placed in the bypass, and Case C included the host vessel and the bypass with a stent and insert placed in the bypass. The blood was considered as a non-Newtonian fluid with the Carreau model, and the flow through it was pulsatile. Furthermore, all the results extracted from this study were taken from the 4th cycle of the simulations to ensure accuracy and robustness. The following main conclusions can be drawn from the present study:

- At the distance of 1 mm after anastomosis and a time of 0.25 s (at systole), there was symmetry in Cases A and B, while in Case C, the symmetry was lost and the axial velocity were increased 18% and 23% compare to Case B and A respectively. Moreover, the strength of the vortices increased compared to Cases A and B, and they were closer to the wall. This process was repeated in diastole at 0.41 s.
- At t = 0.25 s (systole) and 50 mm after the anastomosis, the axial velocity was symmetric in Cases A and B with little fluctuation. Case C broke the symmetry with the maximum velocity shifted from the central to the corner position. The same type of pattern was observed in the secondary velocity field.
- At the location of the stent and t = 0.25 s (systole), velocity in Case C was 25% and 11% higher than in Cases A and B, respectively. Secondary velocity and vortices’ strength were higher and low-velocity zones near the wall were lower. The same was observed at diastole (t = 0.41 s).
- At t = 0.25 s (systole), the WSS in Case C was much higher than the other two cases, and its maximum value reached 113 Pa at host vessel, which was 94% and 59% higher than Cases A and B, respectively.
- In OSI, the anastomosis had the most changes. Moreover, in Case C, around the host vessel, there are the most OSI changes compared to Cases A and B.

As a future study, machine learning-based optimization algorithms such as Physics-Informed Neural Networks (PINN))^50–52^ can also be used to simulate numerous scenarios in a short space of time to obtain optimum configurations to improve hemodynamics for bypass surgeries and other similar clinical interventions. In addition, to assess the deliverability, safety, and long-term patency of the proposed spiral-inducing stent, future work should focus on in vitro testing of the prototype and later on clinical trials.

## Electronic supplementary material

Below is the link to the electronic supplementary material.

Supplementary Material 1

## Data Availability

The data that supports the findings of this study are available within the article and its supplementary material.
