# Supplementary material for: A novel vascular stent and insert concept to improve hemodynamics and support vascular health
Source: Sci Rep. 2025 Jul 8;15:24487. doi: 10.1038/s41598-025-09613-8 (PMC12238337; doi:10.1038/s41598-025-09613-8)
Supplement: Supplementary file 1 — Supplementary Material 1 [file 41598_2025_9613_MOESM1_ESM.docx]

**Supplementary Information**

**A Novel Vascular Stent and Insert Concept to Improve Hemodynamics and Support Vascular Health**

Bahram Vaziri^1^, Saadat Zirak^1*^, Mohammad Azadi^1*^, Amir Keshmiri^2,3*^, Nima Shokri^4^

*^1^Faculty of Mechanical Engineering, Semnan University, Semnan, Iran*

*^2^School of Engineering, The University of Manchester, Manchester, United Kingdom*

*^3^Manchester University NHS Foundation Trust, Manchester, M13 9PL, UK*

*^4^Institute of Geo-Hydroinformatics, Hamburg University of Technology, Hamburg, Germany*

**Corresponding authors, Emails:* *[s_zirak@semnan.ac.ir](mailto:s_zirak@semnan.ac.ir);* *[m_azadi@semnan.ac.ir](mailto:m_azadi@semnan.ac.ir);* *[a.keshmiri@manchester.ac.uk](mailto:a.keshmiri@manchester.ac.uk)*

The results at t = 0.41 s (at diastole) are presented in this supplementary document.


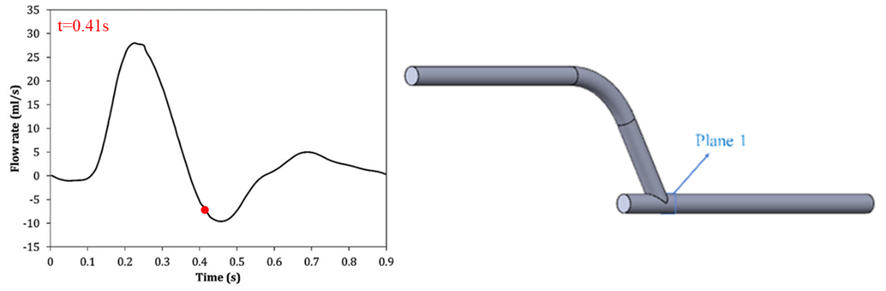


|  |  | Axial Velocity (m/s) |  | Secondary Velocity (m/s) |
| --- | --- | --- | --- | --- |
| Case A | 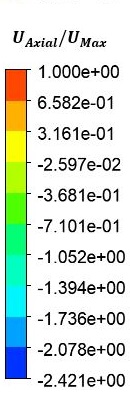 | 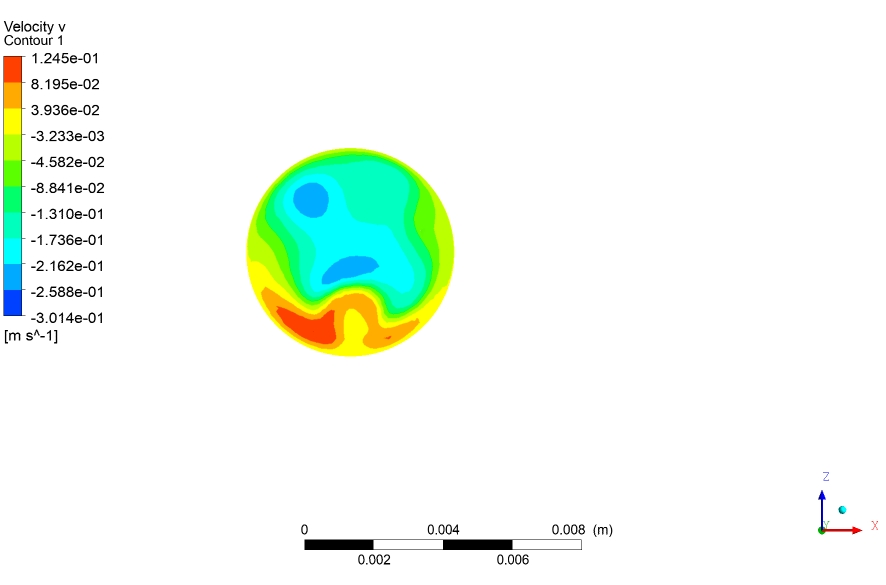 | 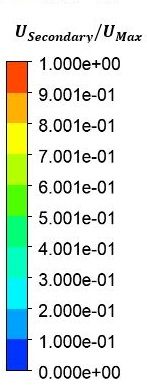 | 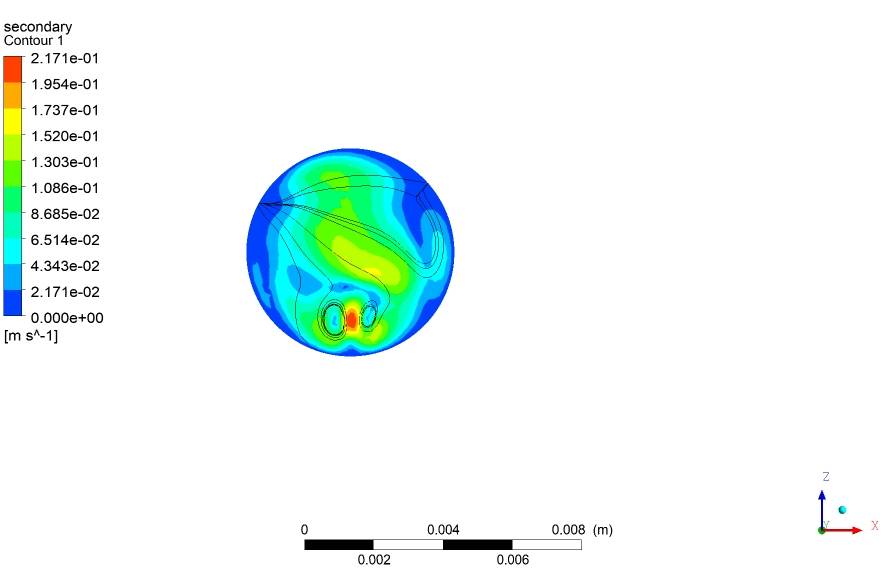 |
| Case B |  | 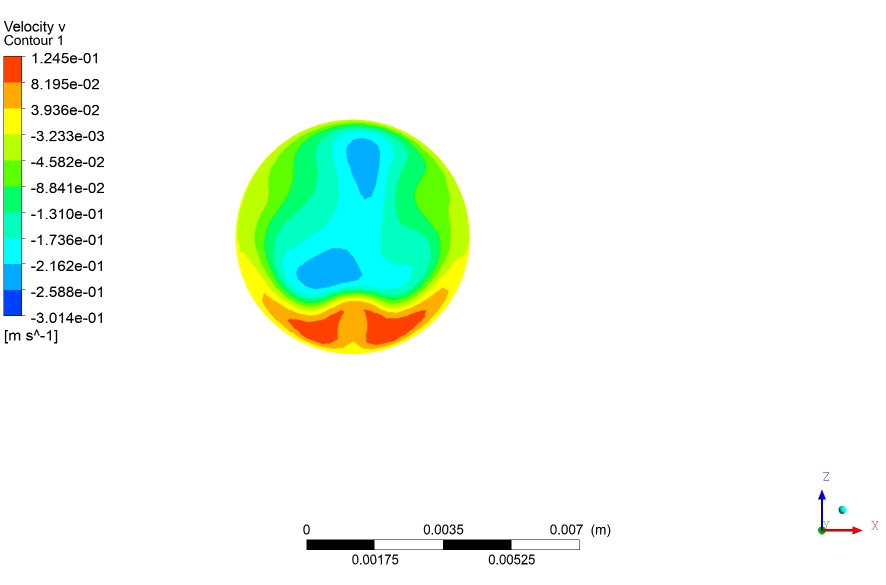 |  | 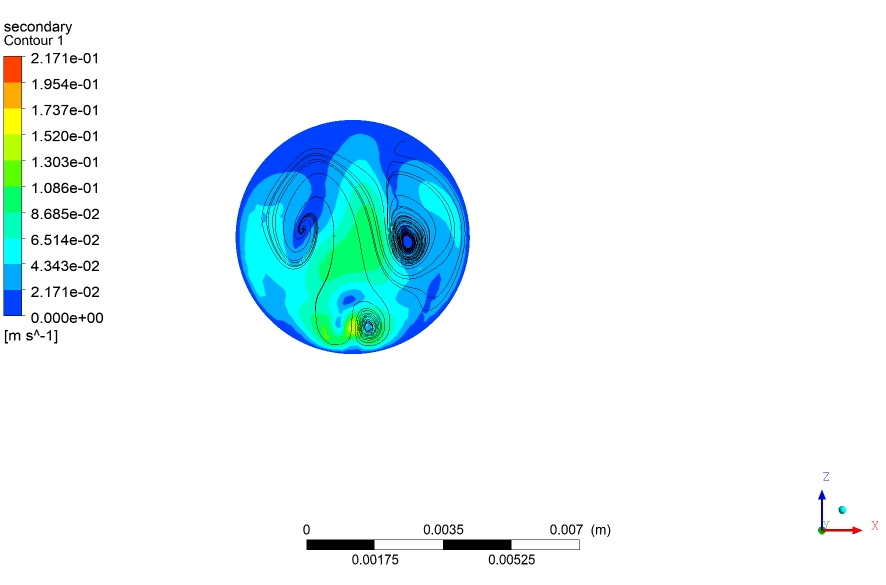 |
| Case C |  | 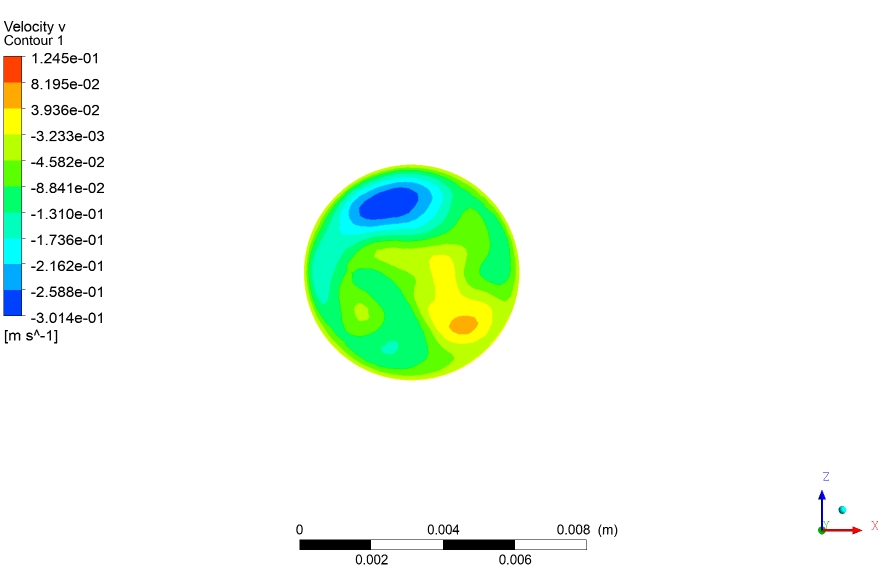 |  | 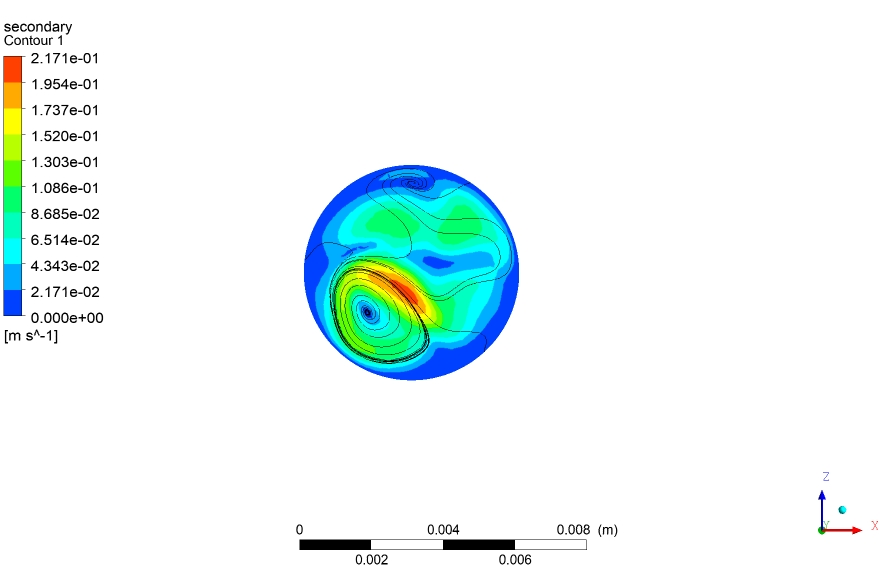 |

Figure S1. The comparison of the axial and secondary velocity at t = 0.41 s and at a distance of 1 mm from the bypass for all three cases


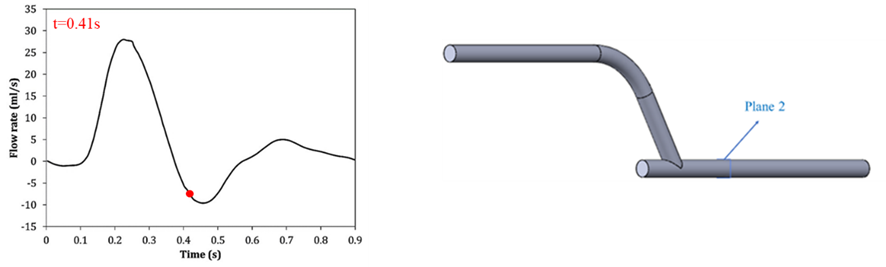


|  |  | Axial Velocity (m/s) |  | Secondary Velocity (m/s) |
| --- | --- | --- | --- | --- |
| Case A | 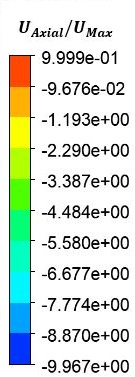 | 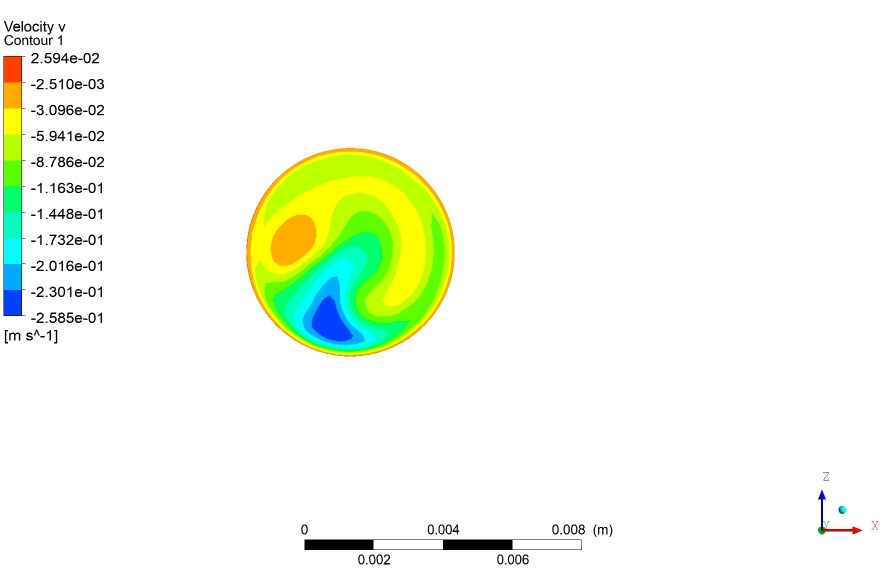 | 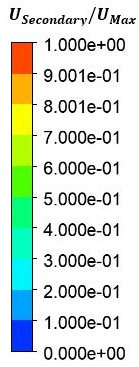 | 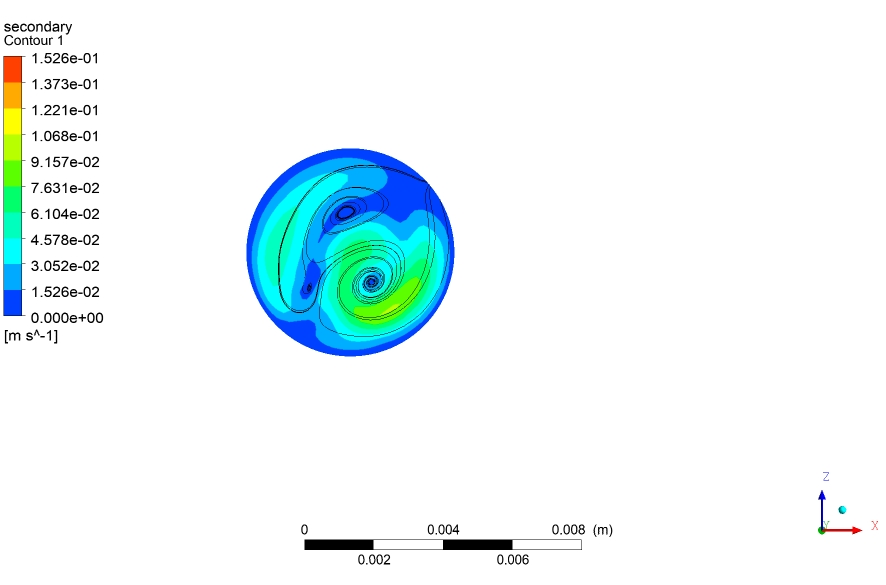 |
| Case B |  | 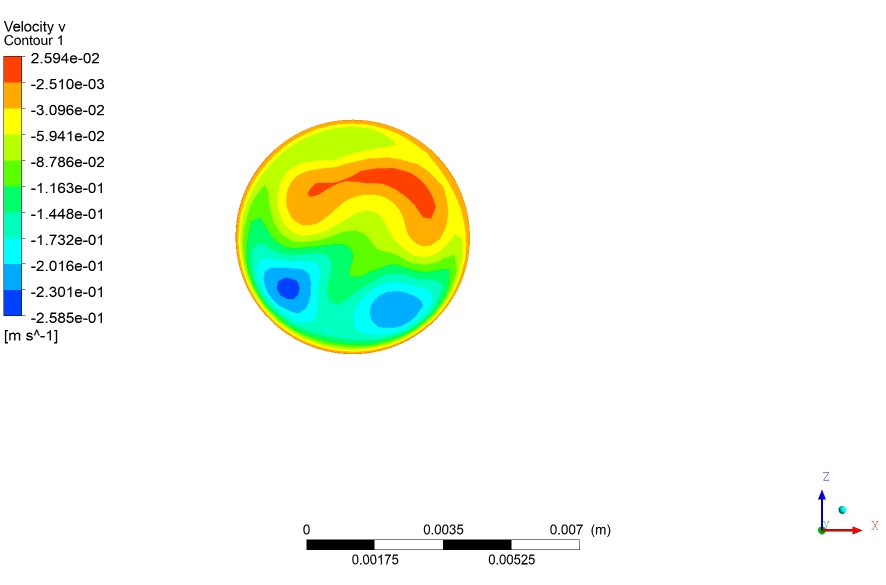 |  | 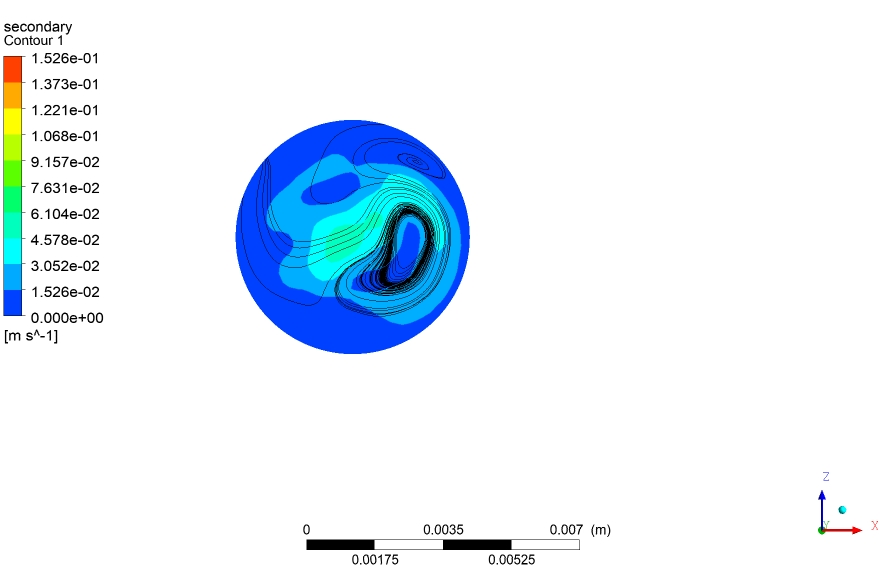 |
| Case C |  | 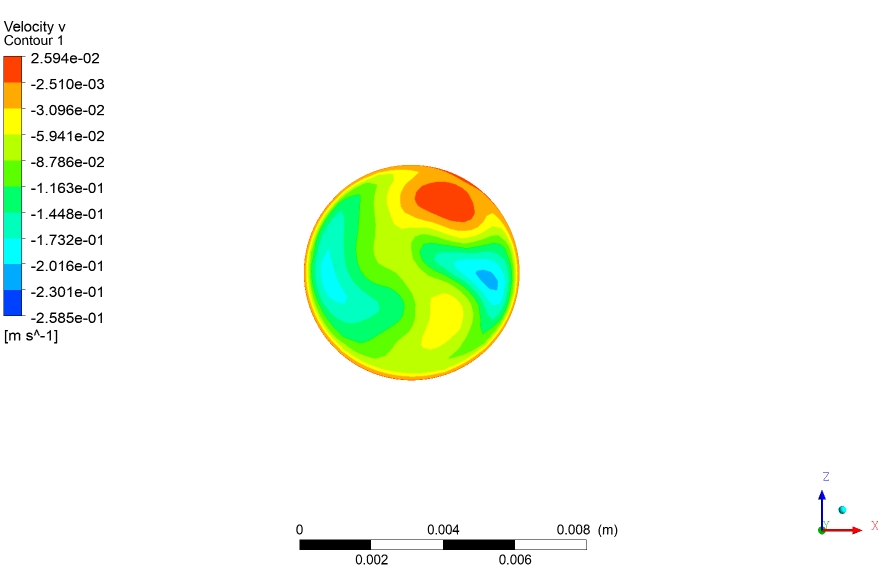 |  | 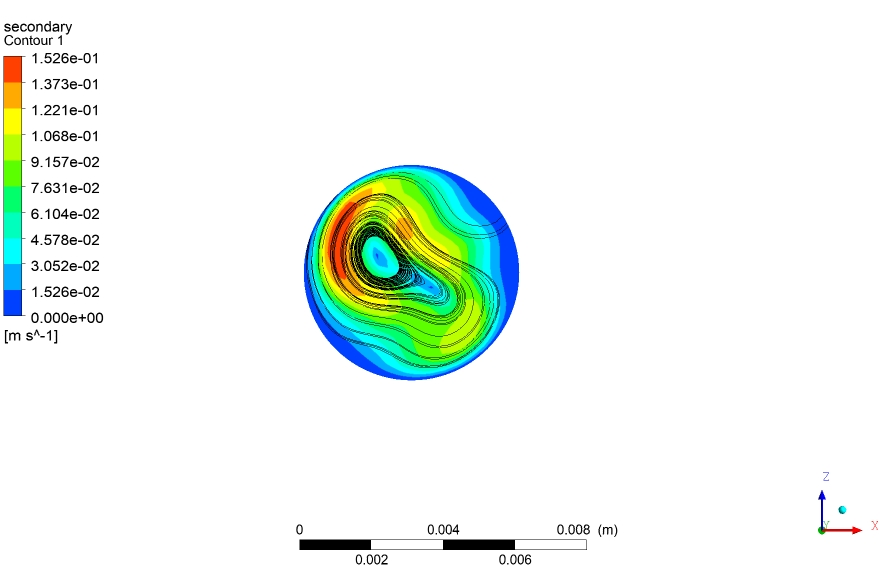 |

Figure S2. The comparison of the axial and secondary velocity at t = 0.41 s and at a distance of 50 mm from the bypass for all three cases


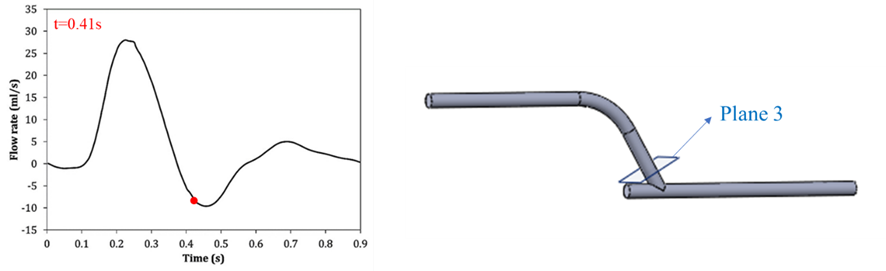


|  |  | Axial Velocity (m/s) |  | Secondary Velocity (m/s) |
| --- | --- | --- | --- | --- |
| Case A | 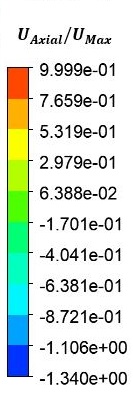 | 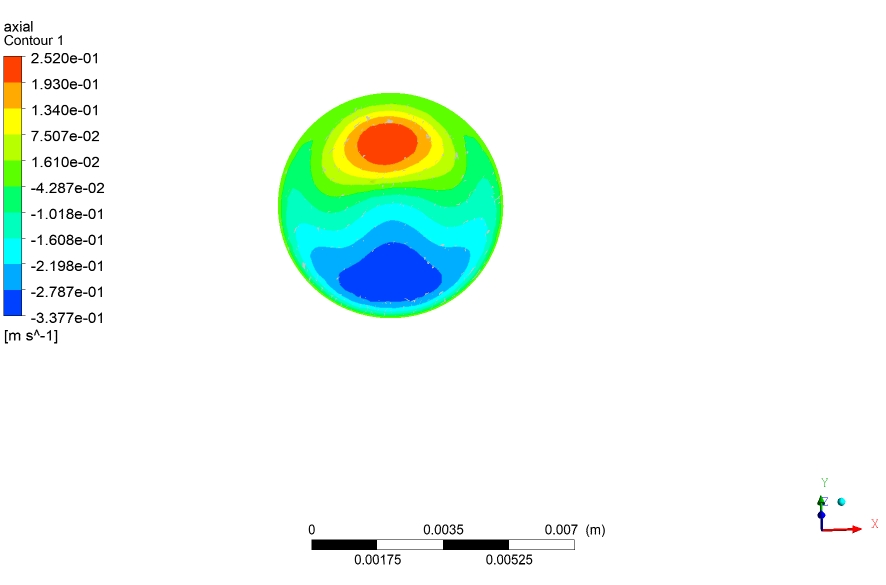 | 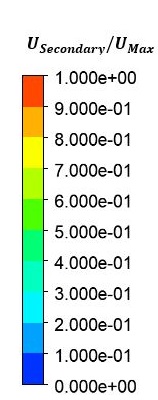 | 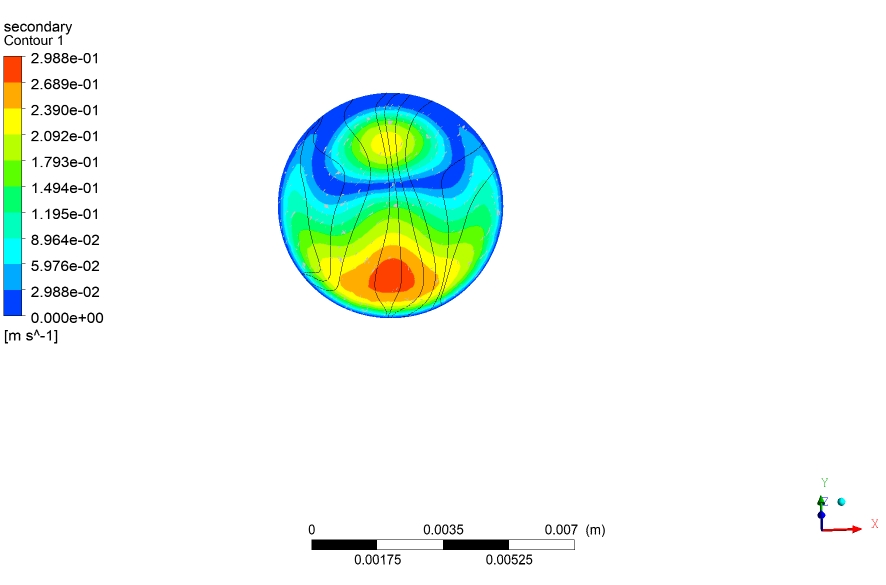 |
| Case B |  | 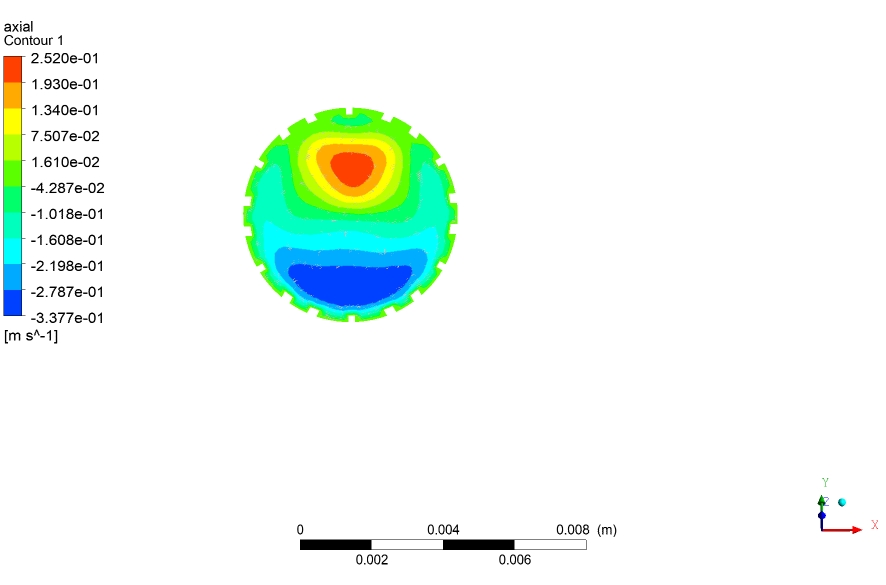 |  | 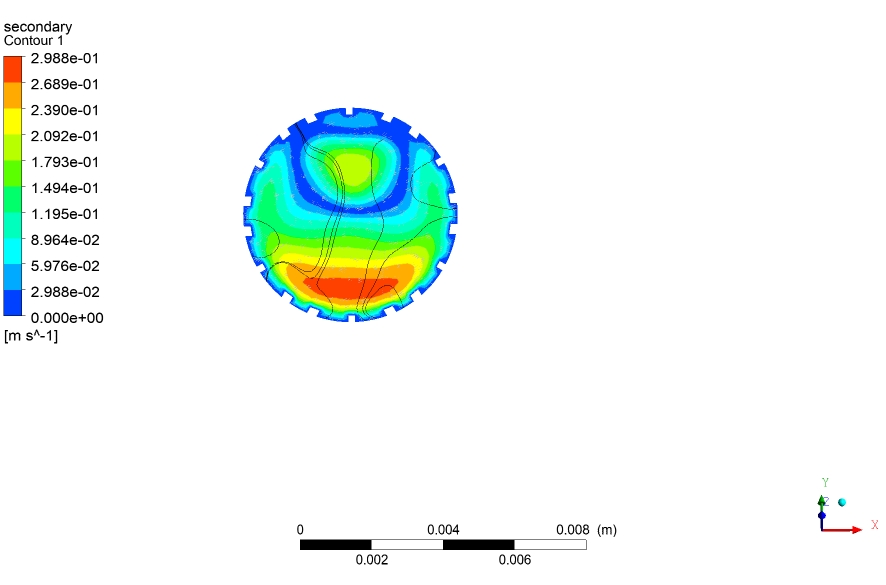 |
| Case C |  | 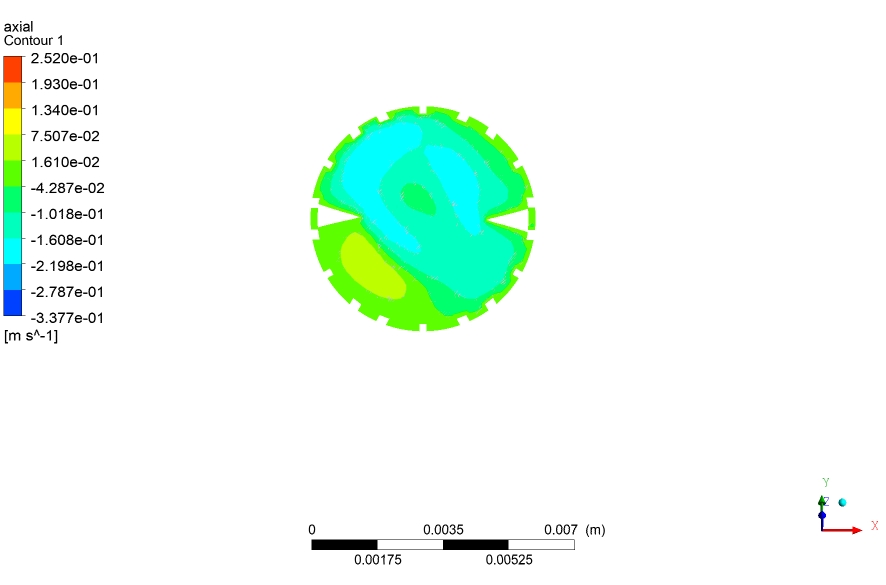 |  | 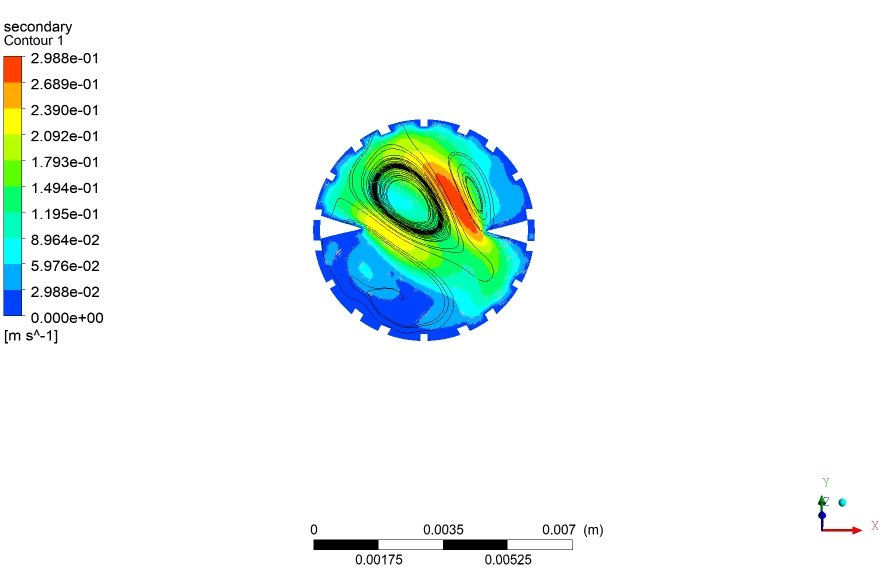 |

Figure S3. The comparison of the axial and secondary velocity at t = 0.41 s and at normal to bypass for all three cases

Table S1. Comparison of axial velocity in different modes at t=0.41 s

| Plans |  | Min  Axial Velocity (m/s) | Max  Axial Velocity (m/s) | Average  Axial Velocity (m/s) |
| --- | --- | --- | --- | --- |
| 1 mm after anastomosis | Case A | -0.24 | 0.12 | -0.09 |
|  | Case B | -0.24 | 0.12 | -0.09 |
|  | Case C | -0.30 | 0.05 | -0.09 |
| 50 mm after anastomosis | Case A | -0.25 | 0 | -0.09 |
|  | Case B | -0.23 | 0.09 | -0.09 |
|  | Case C | -0.21 | 0.02 | -0.09 |
| Normal to bypass | Case A | -0.33 | 0.25 | -0.09 |
|  | Case B | -0.33 | 0.22 | -0.09 |
|  | Case C | -0.21 | 0.06 | -0.1 |

Table S2. Comparison of secondary velocity in different modes at t=0.41 s

| Position |  | Min  Secondary Velocity (m/s) | Max  Secondary Velocity (m/s) | Average  Secondary Velocity (m/s) |
| --- | --- | --- | --- | --- |
| 1 mm after anastomosis | Case A | 0 | 0.21 | 0.07 |
|  | Case B | 0 | 0.14 | 0.04 |
|  | Case C | 0 | 0.21 | 006 |
| 50 mm after anastomosis | Case A | 0 | 0.09 | 0.03 |
|  | Case B | 0 | 0.05 | 0.01 |
|  | Case C | 0 | 0.15 | 0.07 |
| Normal to bypass | Case A | 0 | 0.28 | 0.12 |
|  | Case B | 0 | 0.28 | 0.12 |
|  | Case C | 0 | 0.30 | 0.10 |


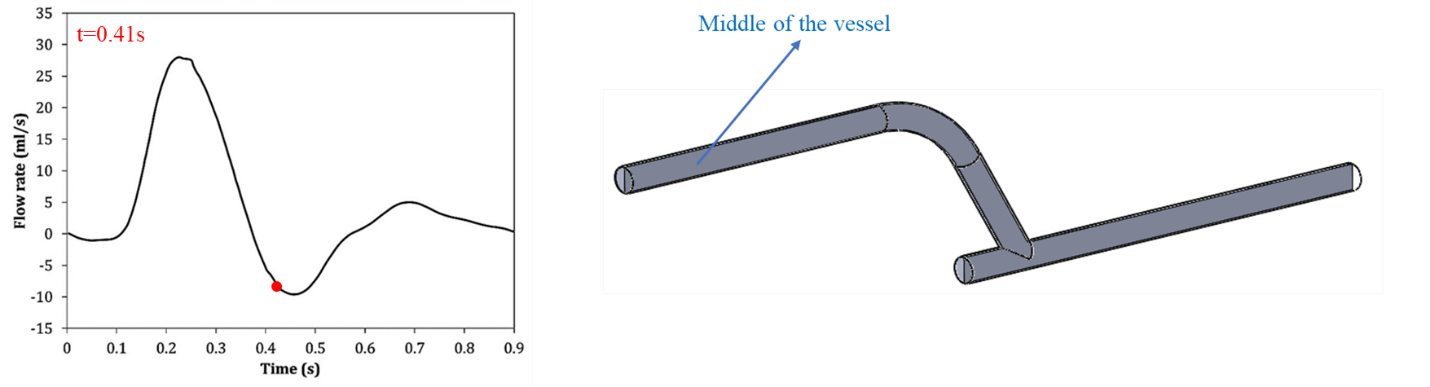


| 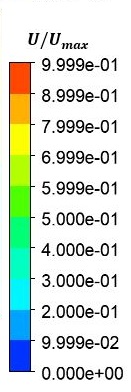 |  | 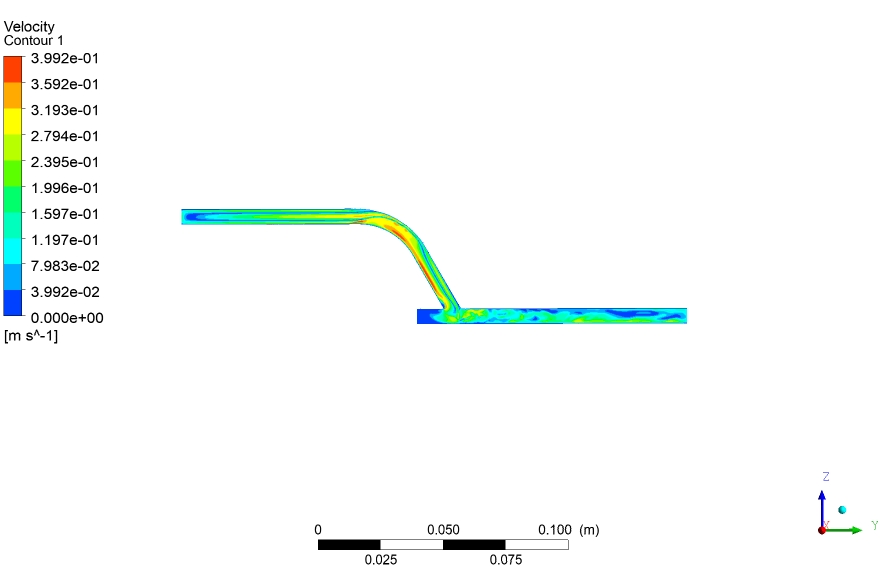 |
| --- | --- | --- |
|  |  | Case A |
|  |  | 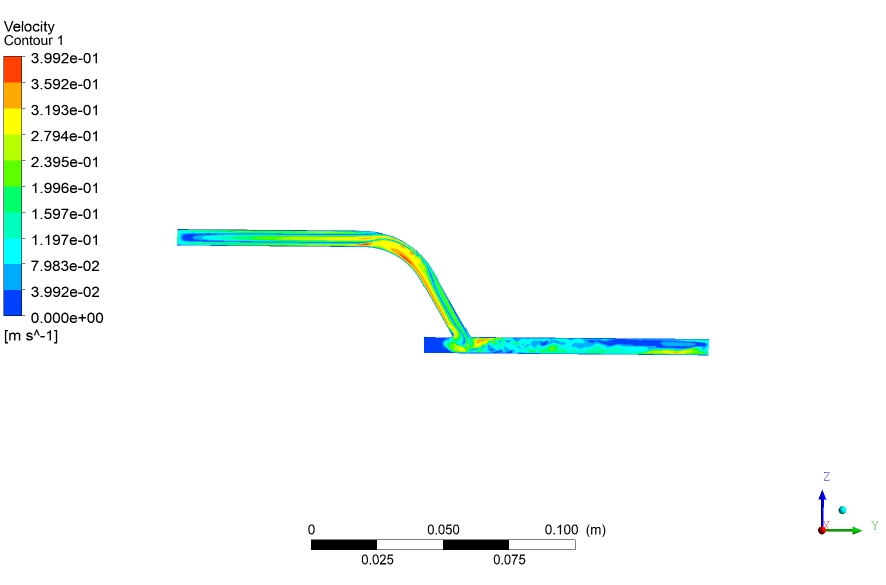 |
|  |  | Case B |
|  |  | 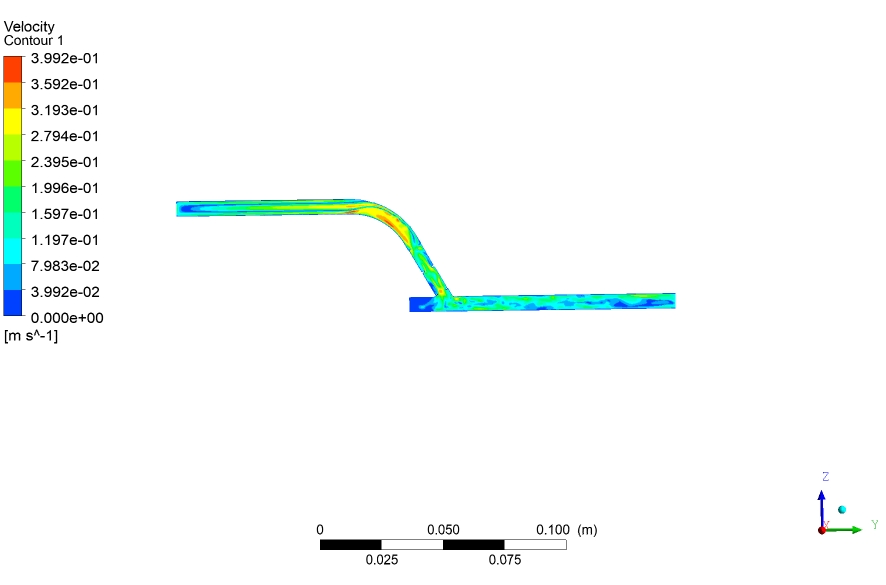 |
|  |  | Case C |

Figure S4. The velocity contour in the middle of the vessel at t = 0.41 s

| 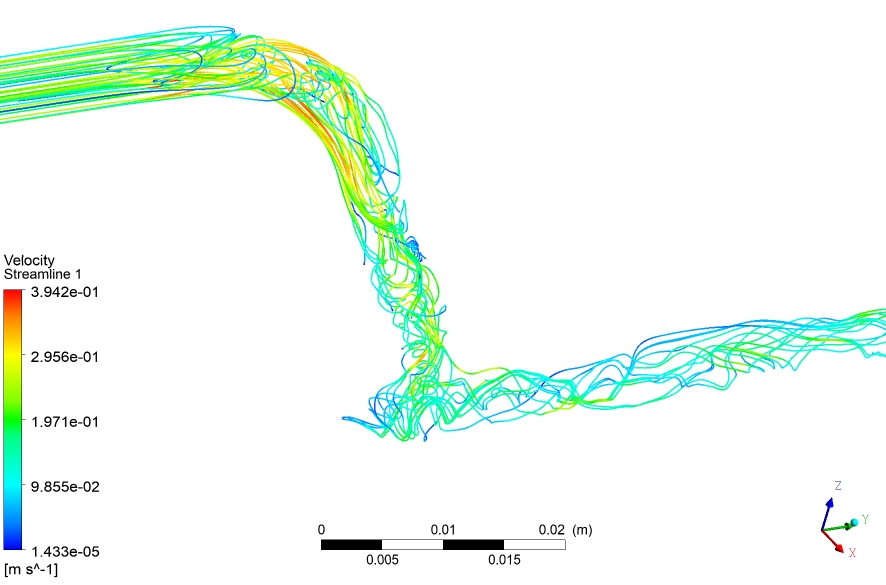 | 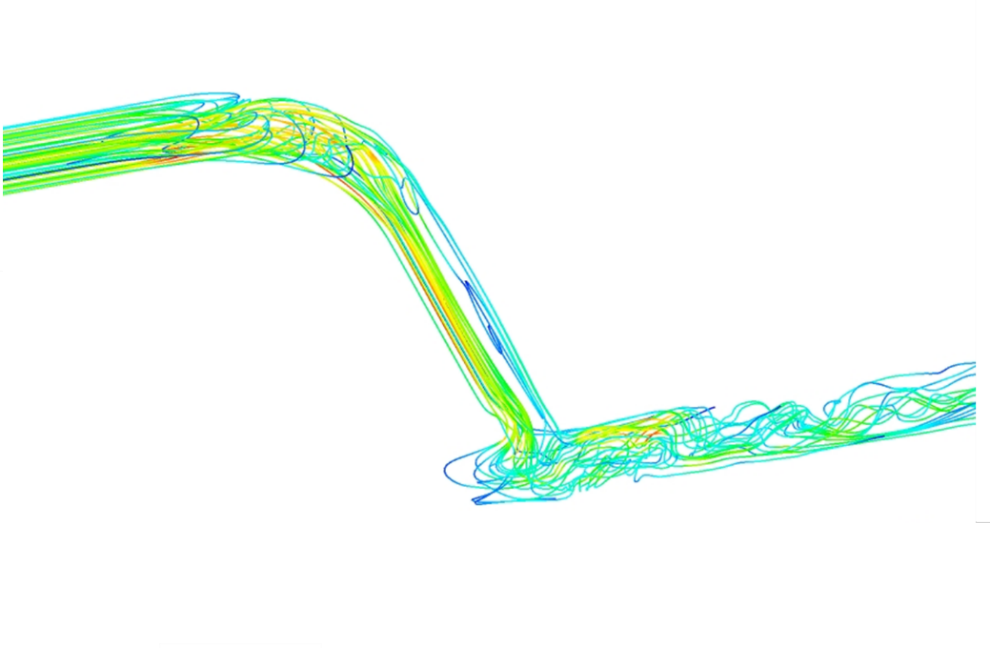 |
| --- | --- |
|  | Case A |
|  | 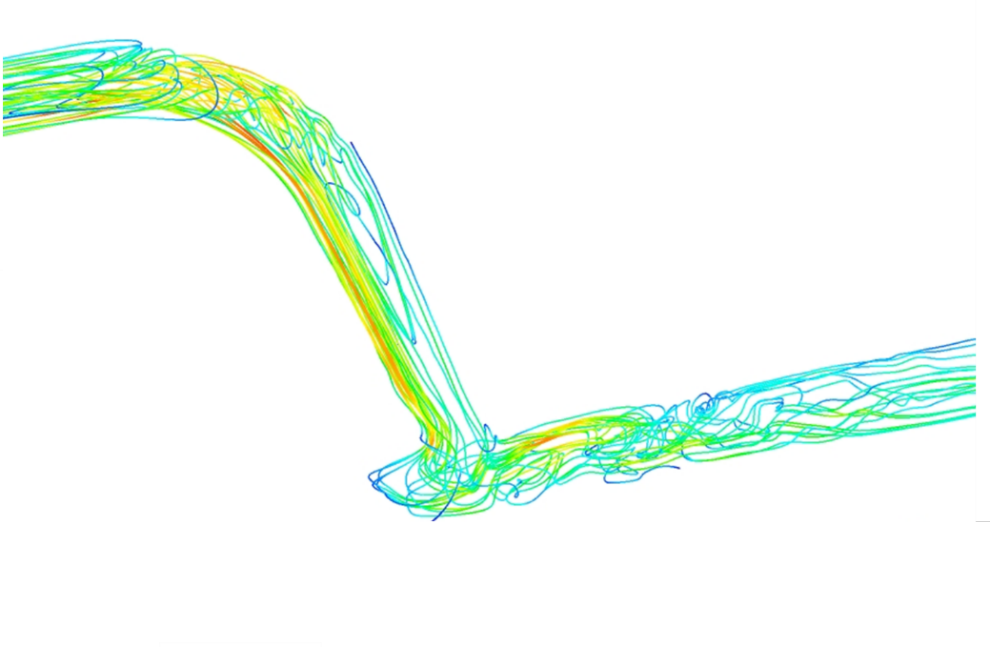 |
|  | Case B |
|  | 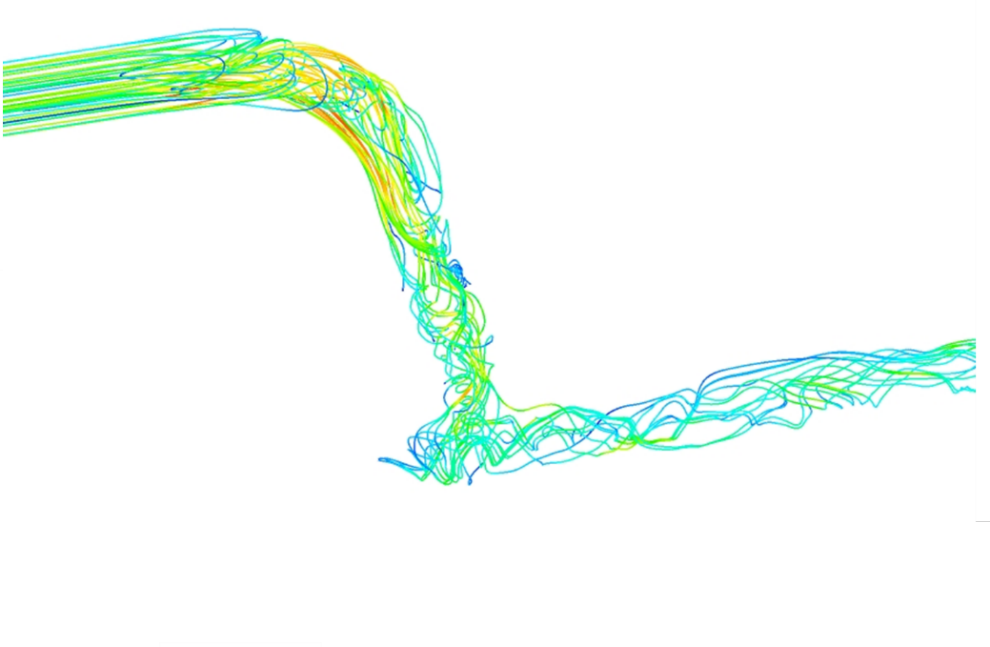 |
|  | Case C |

Figure S5. The streamlines velocity in the bypass area at t = 0.41 s


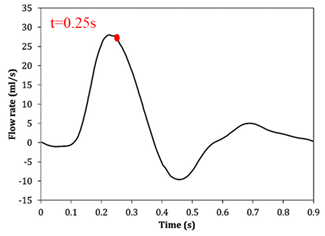


| 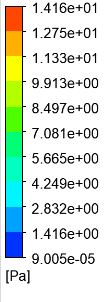 | 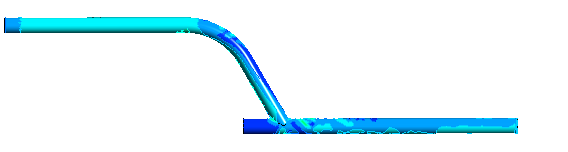 |
| --- | --- |
|  | Case A |
|  | 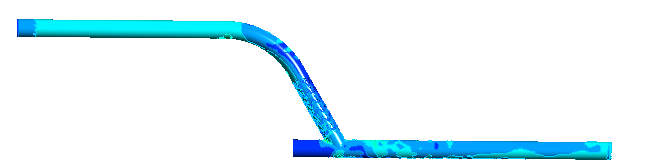 |
|  | Case B |
|  | 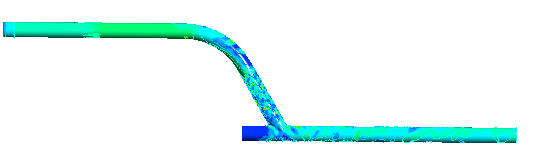 |
|  | Case C |

Figure S6. The WSS contours at t = 0.41 s
